# Investigating the case of human nose shape and climate adaptation

**DOI:** 10.1371/journal.pgen.1006616

**Published:** 2017-03-16

**Authors:** Arslan A. Zaidi, Brooke C. Mattern, Peter Claes, Brian McEcoy, Cris Hughes, Mark D. Shriver

**Affiliations:** 1 Intercollege Graduate Degree Program in Genetics, Huck Institute of Life Sciences, The Pennsylvania State University, State College, Pennsylvania, United States of America; 2 Department of Anthropology, Pennsylvania State University, State College, Pennsylvania, United States of America; 3 Department of Electrical Engineering–KU Leuven, ESAT/PSI—UZ Leuven, Leuven, Belgium; 4 Smurfit Institute of Genetics, Trinity College Dublin, Dublin, Ireland; 5 Department of Anthropology, The University of Illinois at Urbana-Champaign, Illinois, United States of America; 6 Carl R. Woese Institute of Genomic Biology, The University of Illinois at Urbana-Champaign, Illinois, United States of America; Georgia Institute of Technology, UNITED STATES

## Abstract

The evolutionary reasons for variation in nose shape across human populations have been subject to continuing debate. An import function of the nose and nasal cavity is to condition inspired air before it reaches the lower respiratory tract. For this reason, it is thought the observed differences in nose shape among populations are not simply the result of genetic drift, but may be adaptations to climate. To address the question of whether local adaptation to climate is responsible for nose shape divergence across populations, we use Qst–Fst comparisons to show that nares width and alar base width are more differentiated across populations than expected under genetic drift alone. To test whether this differentiation is due to climate adaptation, we compared the spatial distribution of these variables with the global distribution of temperature, absolute humidity, and relative humidity. We find that width of the nares is correlated with temperature and absolute humidity, but not with relative humidity. We conclude that some aspects of nose shape may indeed have been driven by local adaptation to climate. However, we think that this is a simplified explanation of a very complex evolutionary history, which possibly also involved other non-neutral forces such as sexual selection.

## Author summary

The study of human adaptation is essential to our understanding of disease etiology. Evolutionary investigations into why certain disease phenotypes such as sickle-cell anemia and lactose intolerance occur at different rates in different populations have led to a better understanding of the genetic and environmental risk factors involved. Similarly, research into the geographical distribution of skin pigmentation continues to yield important clues regarding risk of vitamin D deficiency and skin cancer. Here, we investigate whether variation in the shape of the external nose across populations has been driven by regional differences in climate. We find that variation in both nares width and alar base width appear to have experienced accelerated divergence across human populations. We also find that the geospatial distribution of nares width is correlated with temperature, and absolute humidity, but not with relative humidity. Our results support the claim that local adaptation to climate may have had a role in the evolution of nose shape differences across human populations.

## Introduction

The shape of the nose, like many other parts of the face, varies both within as well as across human populations. For example, the distance between the nasal alare (wings of the nose) are significantly larger in individuals of West African, South Asian, and East Asian ancestry as compared to persons with European ancestry [1]. The nasal index (width/height of the nasal aperture of the skull) is also known to vary significantly among populations [1,2]. Whether these population differences in nose shape are due primarily to genetic drift or natural selection is unclear.

A vital function of the nose is to warm inspired air to core body temperature and saturate it with water vapor before it reaches the lower respiratory tract [3]. In fact, inhaled air reaches 90% of the required temperature and humidity levels before even reaching the nasopharynx, implicating the nasal cavity as the major conditioning apparatus in the respiratory tract [4,5]. This conditioning acts to maintain proper functioning of the mucociliary apparatus, which functions in trapping particles and pathogens and removing them from the airways. Low humidity in the respiratory tract leads to impaired mucociliary function and increases the risk of both upper and lower respiratory tract infections [6]. Much of the air conditioning occurs as it passes through the turbinates, the walls of which are lined with blood vessels and mucus producing goblet cells [4]. Studies have shown that the efficacy of the conditioning process depends on the flow dynamics of the inspired air, which in turn, depend on the geometry of the nasal cavity and inlets [5,7]. Because of the function of the nose as an air-conditioning apparatus, it is hypothesized that differences in nose shape across populations may have been driven by local adaptation to climate [2,3,8].

There are several challenges in testing this hypothesis. We know there is substantial nose shape variation among human populations, in both the external morphology of the nose as well as the underlying cranial morphology [1,2,9]. While this can be explained by adaptation to local selection pressures, it could also be explained by the fact that phenotypic differences among geographically distant populations can arise simply due to genetic drift. Thus, in order to invoke divergent selection as an explanation, one must demonstrate that the observed variation in nose shape across human populations is greater than that expected under genetic drift alone. This can be carried out using the Qst statistic, which measures the degree of genetic differentiation underlying a quantitative trait [10]. In principle, the Qst of a neutrally evolving trait is expected to follow the Fst distribution of neutrally evolving loci [11]. Thus, when Qst is much greater than Fst, trait divergence exceeds neutral expectations and can be attributed to divergent selection [11]. The problem with Qst is that its calculation requires knowledge of the additive genetic variances within- and among-populations. These can only be estimated reliably using ‘common-garden’ experiments, in which environmental effects on the phenotype can be effectively controlled. Since such experiments are not possible in humans, Qst-based inference regarding divergent selection on human phenotypes necessitates making realistic assumptions about the heritabilities of the phenotypes in question.

Several studies have used this approach and found that while most aspects of the skull seem to be evolving neutrally, the shape of the nasal aperture appears to be more differentiated across human populations than expected under genetic drift [9,12,13]. More recently, it was also reported that the divergence in the shape of the external nose, at least between Europeans and Han Chinese populations, also exceeds neutral expectations [14]. While this might be true, most of these studies employed anticonservative heritability assumptions, which overestimate the genetic differentiation underlying a trait, and thus, lead to incorrect conclusions regarding the relative roles of selection and drift in driving phenotypic differences among populations.

Here, we carried out an exploration of external nose shape variation in light of quantitative genetic theory to investigate whether nose shape variation among populations exceeds neutral expectations. In doing so, we discuss the methodological challenges involved in tackling such questions in humans, and show how the limitations of previous studies can be addressed with recent advances in statistical genetics. As proof of principle, we compare the differentiation of nose shape with two highly heritable morphological traits, which are known to have a polygenic basis; namely height and skin pigmentation [15–18]. Both height and skin pigmentation exhibit substantial variation within and across human populations, and are thought to be under positive selection in various populations [19–22]. Skin pigmentation is also a good example of a phenotype which is known to have evolved in response to a geospatially varying selection pressure: exposure to ground level ultraviolet B radiation (UVB) [23]. Finally, we test whether clinal variation in aspects of nose shape, which appear to be under accelerated divergence across populations, covaries with geographic variation in temperature and humidity, in order to determine whether this divergence is due to climatic selection pressures.

## Results

### Describing variation in nose shape

To quantify variation in nose shape, we first captured high resolution 3D images of participants’ faces using the 3dMD Face system (*3dMD Atlanta*, *GA*). Five positioning landmarks (two on the inner corner of the eyes, two on the outer corners of the mouth, and one on the tip of the nose) were placed in order to establish facial orientation. A spatially dense mesh of 7,150 quasi-landmarks (QLs) was mapped onto each image and its reflection. Generalized Procrustes Superimposition [24] was carried out on both sets of images (original and reflected) to remove differences in position and orientation. The Procrustes coordinates of the original and reflected image for each participant were then averaged to remove effects of bilateral asymmetry following Claes *et al*. (2014) [25]. The nose region, which is comprised of 709 out of the 7,150 QLs, was selected for downstream analyses.

**Fig 1:**
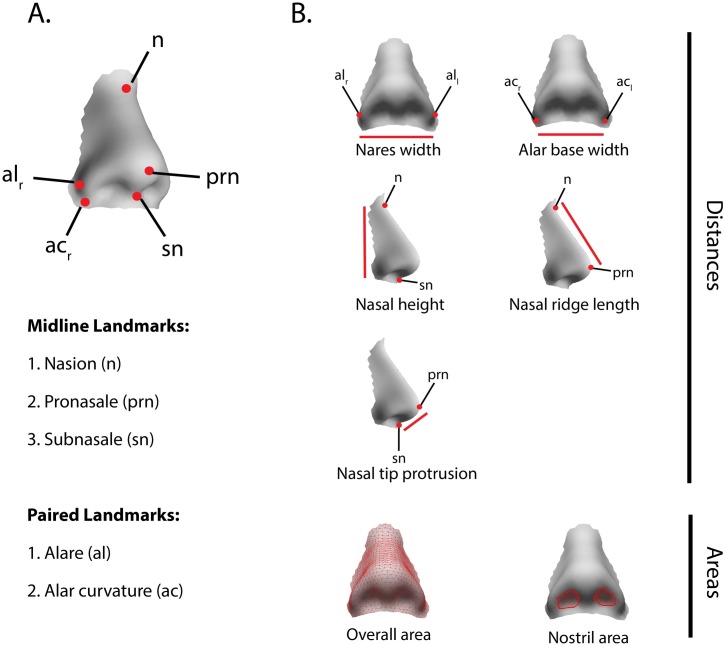
Measurements used to describe the shape of the nose. A) The locations of landmarks, which were used to calculate linear distances, are shown as red points. B) Five Linear distances (red lines) and two surface areas (red mesh) were used to describe nose shape. Linear distances were calculated from the 3D coordinates of landmarks (red points). Surface areas were computed as the sum of the polygons highlighted in red.

We used linear distances and areas to characterize the shape of the nose (Fig 1). The linear distances (measured in mm) were calculated using seven standard anthropometric landmarks: i. nasion (n), ii. pronasale (prn), iii. subnasale (sn), iv. Left alar curvature, (al_l_), v. right alar curvature (al_r_), vi. left alar base (ac_l_), and vii. Right alar base (ac_r_) (Fig 1) [26,27]. These seven landmarks were placed on the spatially-dense mesh of 709 QLs, which facilitated automated placement on each face. We used the x, y, and z coordinates of these landmarks to calculate five Euclidean distances: i. nares width (al_l_−al_r_), ii. alar base width (ac_l_−ac_r_), iii. nasal height (n–sn), iv. nasal ridge length (n–prn), and v. nasal tip protrusion (sn–prn) (Fig 1). We also computed two areas (measured in mm^2^): i. total external area of the nose and ii. mean nostril area (Fig 1) (Methods).

**Fig 2:**
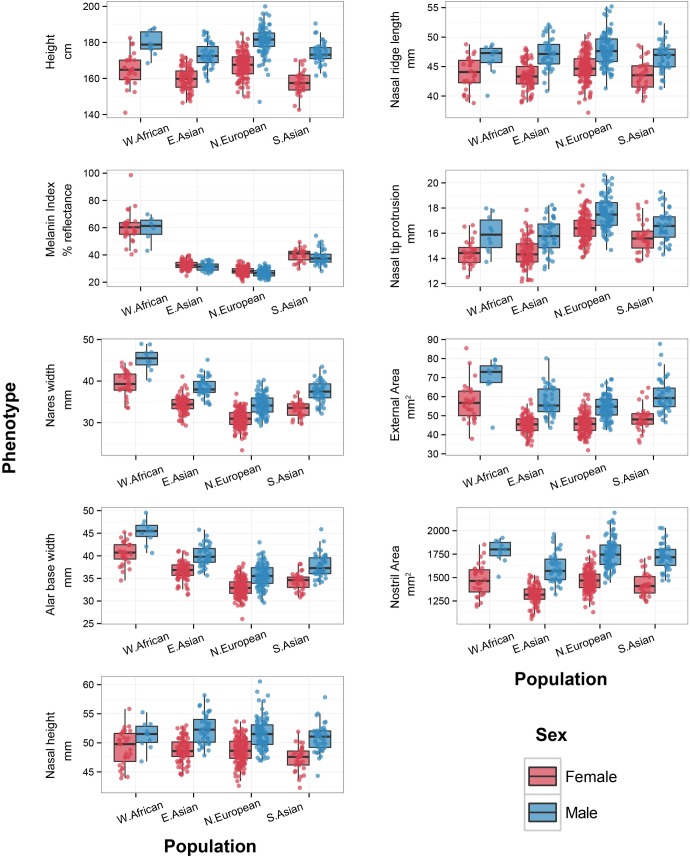
Boxplots of phenotypes by population and sex overlaid with the individual data points. Height is measured in centimeters and melanin index is measured in percentage reflectance (Methods). Linear distances are measured in millimeters (mm) and area are measured in mm^2^. Points are individual observations and the color of the boxplots and points represents sex with blue indicating males and red indicating females.

Fig 2 shows the distribution of aspects of nose shape in males and females from four human population groups: West African, East Asian, South Asian, and Northern European. These groups were defined based on genetic and self-reported ancestry (Methods). Fig 2 also shows the distribution of height and melanin index, a measure of skin pigmentation level derived from reflectance spectrophotometry (measured on the upper inner arms; see Methods), for comparison. The sample size, mean, and standard deviation for each phenotype are provided in Table 1. One clear observation is that all aspects of nose shape, at least those considered here, are highly sexually dimorphic (Fig 2, Table 1). Males, on average, tend to have wider nares, longer nasal ridges, more outwardly protruding nasal tips, bigger nostrils, and a larger overall external surface area, compared to females (Fig 2, Table 1). All nasal measurements are also significantly different across populations (S1 Table). Based on Table 1, we note some general patterns. For example, nares width and alar base width are largest in W. Africans and smallest in N. Europeans. These findings are consistent with previous observations [1,2]. Nasal tip protrusion is greatest in N. Europeans and smallest in W. Africans and E. Asians. E. Asians also tend to have the smallest noses in terms of external surface area.

**Table 1:** Sample size, mean and standard deviation of phenotypes by sex and population.

Population	Sex	N^a^	Height (cm)	Melanin Index (% reflectance)	Nares Width (mm)	Alar base width (mm)	Nasal height (mm)	Nasal ridge length (mm)	Nasal tip protrusion (mm)	External surface area (mm)	Nostril area (mm)
W. African	Male	10, 6, 10	179.89^b^ (6.48)	59.23 (9.68)	45.36 (2.74)	45.31 (2.61)	51.31 (2.36)	46.31 (2.63)	15.94 (1.44)	1782.13 (122.92)	70.17 (10.41)
W. African	Female	30, 28, 30	164.68 (8.61)	60.27 (11.34)	39.50 (2.95)	40.57 (2.56)	49.15 (3.04)	43.82 (2.76)	14.43 (0.98)	1477.42 (167.39)	57.18 (9.75)
E. Asian	Male	43, 33, 43	173.02 (6.79)	31.42 (2.94)	38.55 (2.28)	39.98 (2.31)	52.35 (2.52)	47.14 (2.59)	15.81 (1.35)	1604.73 (154.02)	57.59 (7.61)
E. Asian	Female	84, 74, 84	159.94 (6.23)	32.63 (2.94)	34.35 (2.02)	36.60 (1.93)	48.76 (2.03)	43.41 (2.24)	14.47 (1.11)	1315.35 (100.95)	45.40 (4.77)
N. European	Male	91, 91, 91	181.00 (7.72)	26.71 (2.68)	34.29 (2.42)	35.66 (2.53)	51.59 (2.64)	47.82 (2.78)	17.62 (1.25)	1758.72 (153.36)	54.46 (5.97)
N. European	Female	145, 144, 145	167.15 (6.70)	28.19 (2.60)	30.91 (2.07)	33.00 (1.98)	48.62 (2.11)	44.78 (2.37)	16.43 (1.02)	1470.61 (118.68)	45.80 (5.13)
S. Asian	Male	42, 35, 42	173.91 (6.13)	38.50 (5.82)	37.47 (2.59)	37.77 (2.57)	50.81 (2.34)	46.59 (2.35)	16.60 (1.23)	1719.14 (134.48)	60.83 (8.87)
S. Asian	Female	31, 26, 31	157.74 (6.72)	39.89 (4.72)	33.20 (1.89)	34.33 (1.92)	47.31 (2.13)	43.57 (2.47)	15.68 (1.26)	1422.48 (122.86)	48.52 (6.48)

a. Sample size for height, melanin index, and nose shape measurements, respectively.

b. The first number is the mean and the number in parenthesis is the standard deviation.

### Testing for accelerated divergence

Differences in a phenotype can accumulate across populations simply due to genetic drift. In order to invoke positive directional selection, one must demonstrate that the variation across populations is more than that expected under genetic drift. We used Qst (see Leinonen *et al*. [28] for a recent review), the quantitative genetic analog of Fst [10,29], to test whether certain aspects of nose shape exhibit greater differentiation across populations than expected under genetic drift alone. Qst measures the degree of genetic differentiation in a quantitative trait across populations and is defined as:

$$\mathrm{Qst}=\frac{\sigma_{gb}^{2}}{\sigma_{gb}^{2}+{2\sigma }_{gw}^{2}}$$ (1)

where $\sigma_{gb}^{2}$ and $\sigma_{gw}^{2}$ are the components of phenotypic variance due to additive genetic effects among and within populations, respectively. It has been shown that, in principle, the distribution of Qst of a quantitative trait that has evolved under genetic drift alone is expected to be equal to Fst of neutral genetic markers [11,30,31]. This expectation allows one to compare Qst to Fst to test whether genetic drift alone is sufficient to explain the divergence of a trait among populations. If the Qst of a trait across a set of populations is much greater than Fst, it means that the phenotypic differentiation exceeds the expectation under neutrality.

The components of additive genetic variance, $\sigma_{gb}^{2}$ and $\sigma_{gw}^{2}$, are typically estimated from ‘common-garden’ experiments in which the effects due to the environment can be controlled [28]. This is often not possible in non-model organisms, especially humans, because of practical and ethical limitations. In such cases, $\sigma_{gb}^{2}$ and $\sigma_{gw}^{2}$ can be estimated from the among- and within-population components of *phenotypic* variance, $\sigma_{pb}^{2}$ and $\sigma_{pw}^{2}$, respectively, provided the heritabilities underlying these components are known:

$$\mathrm{Qst}=\frac{c\sigma_{pb}^{2}}{c\sigma_{pb}^{2}+2h^{2}\sigma_{pw}^{2}}=\frac{\frac{c}{h^{2}}\sigma_{pb}^{2}}{\frac{c}{h^{2}}\sigma_{pb}^{2}+2\sigma_{pw}^{2}}$$ (2)

Here $\sigma_{pb}^{2}$ and $\sigma_{pw}^{2}$ are among- and within-population components of the phenotypic variance and *c* and *h*^*2*^ are proportions of $\sigma_{pb}^{2}$ and $\sigma_{pw}^{2}$, respectively, that are due to additive genetic effects. Both *c* and *h*^*2*^ are can range from 0 (none of the variance is due to additive genetic effects) to 1 (all of the variance is due to additive genetic effects). Eq (2) shows that Qst calculated from phenotypic variance components depends on the ratio between *c* and *h*^*2*^ [32]. Without prior information, it is reasonable to assume *c/h*^*2*^ = 1, i.e., the proportion of phenotypic variance due to additive genetic effects is the same among- and within-populations. Qst calculated this way is sometimes referred to as Pst [33]. However, we will continue to use the term Qst to avoid confusion and will evaluate the validity of the assumption that *c/h*^*2*^ = 1 in the following section.

**Fig 3:**
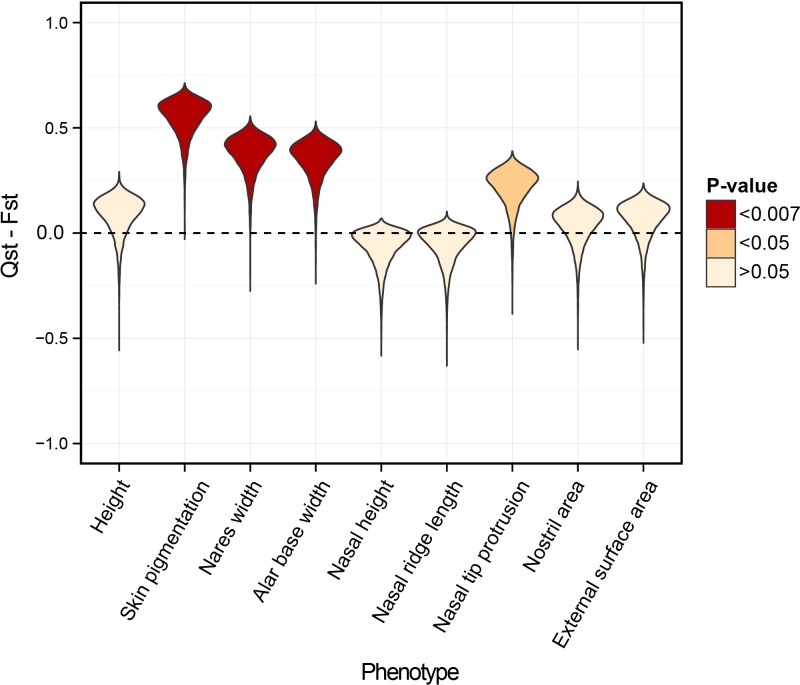
Qst–Fst results across all populations. The bootstrapped distribution of Qst–Fst for each phenotype (shown by a violin plot) is compared against the expected value of zero under neutrality (horizontal dashed line). Phenotypes, which exhibit accelerated divergence (using a Bonferronni corrected p-value threshold of 0.0071), are shown in red.

We calculated Qst for each aspect of nose shape, described in the previous section, across four human population groups: i) West African (N = 40), ii) North European (N = 236), iii) East Asian (N = 127), and iv) South Asian (N = 73) (see Methods for selection criteria). We used a non-parametric bootstrap approach to generate the empirical distributions of Qst and Fst and to test whether the observed value of Qst is greater than Fst (Methods). The statistic we used is Qst–Fst, which, under the null hypothesis of genetic drift, is expected to be equal to zero. The larger the Qst–Fst of a phenotype, the stronger the evidence that the variation in the phenotype across populations is more than that expected under genetic drift alone. We refer to outliers in the neutral distribution as signals of accelerated divergence for brevity. The strength of evidence for accelerated divergence can be measured using an empirical p-value, which is the proportion of bootstrapped values of Qst–Fst that are less than zero. To compare with other quantitative traits with a polygenic basis, we also tested whether height and skin pigmentation exhibit signals of accelerated divergence. The results are illustrated in Fig 3 and the p-values are listed in Table 2. We treat phenotypes that pass a stringent Bonferonni correction (p-value <0.0071 = 0.05/7 for seven nose shape traits) as exhibiting signals of accelerated divergence across populations.

**Table 2:** Results for tests of accelerated divergence across populations.

Phenotype	Qst^c^	empirical p-value^d^	Critical value of c/h^2^ ^e^
Height	0.177	0.1433	>1
Skin pigmentation	0.642	0.0001	0.25
Alare width	0.467	0.0028	0.50
Alar base width	0.440	0.0049	0.55
Nasal height	0.023	0.7886	>1
Nasal ridge length	0.035	0.7053	>1
Nasal tip protrusion	0.306	0.0274	~1
External surface area	0.127	0.2601	>1
Nostril area	0.154	0.1919	>1

c. Qst calculated assuming c/h^2^ = 1.

d. p-value calculated using a non-parametric bootstrap approach (see Methods).

e. critical value of c/h^2^ at which lower 95% bound of Qst meets upper 95% bound of Fst.

As expected, skin pigmentation shows a strong signal of accelerated divergence across populations (Qst = 0.642, p-value = 1.00E-04). This is in accordance with the idea that differences in skin pigmentation, at least across continental populations, have been driven by positive selection. Height, on the other hand, does not show signals of accelerated divergence (Qst = 0.177, p-value = 0.14). While height seems to be under positive *selection within* European and African populations separately [19,34], it does not appear to be driven by divergent selection pressures across the populations considered here. Of all the nose shape measurements, only nares width (Qst = 0.467, p-value = 2.80E-03) and alar base width (Qst = 0.440, p-value = 4.90E-03) exhibit signals of accelerated divergence. However, the degree of divergence in these traits is not as high as that observed for skin pigmentation.

### Evaluating validity of assumptions regarding *c* and *h*^*2*^

**Fig 4:**
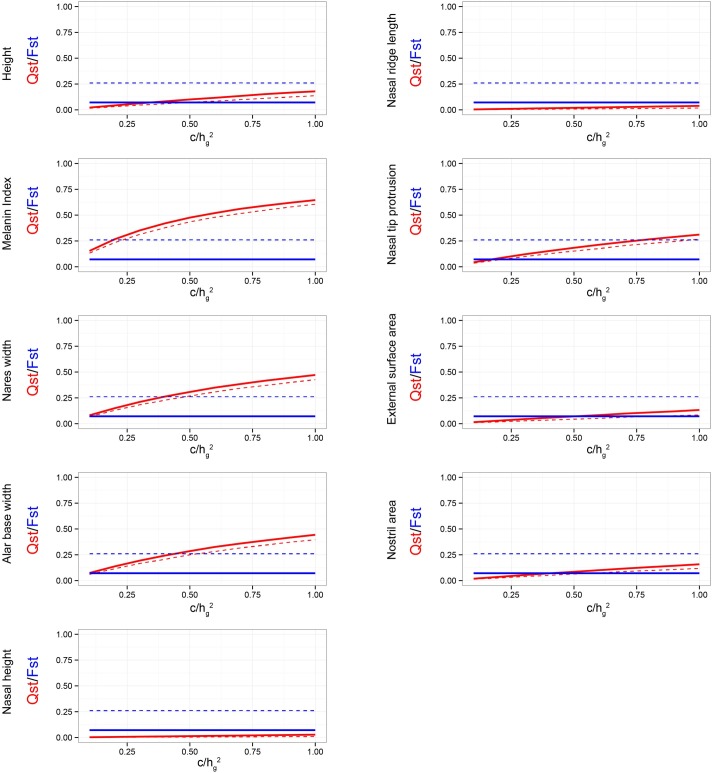
Sensitivity plots showing critical values of *c/h*^*2*^. Change in median Qst of height, skin pigmentation, and nose shape measures as a function of *c/h*^*2*^ is shown as a solid red line. Median Fst is shown as a solid blue line. The lower and upper 95% bounds for Qst and Fst are shown as dashed red and blue lines, respectively. The critical value at which the lower bound of Qst meets the upper bound of Fst is shown on the upper left corner of each plot. Lower critical values indicate the Qst–Fst is more robust to *c/h*^*2*^ assumptions.

We estimated Qst under the assumption that *c/h*^*2*^ = 1. In other words, we assume that the proportion of between-population variance in the trait due to additive genetic effects is the same as the proportion of within-population variance due to additive genetic effects. This can be an incorrect assumption if a large proportion of the phenotypic variance between populations is due to direct environmental effects (i.e., phenotypic plasticity). If the true value of *c/h*^*2*^ is drastically lower than 1, phenotype-derived estimates of the among-population genetic variance, and hence, Qst will be inflated, resulting in false-positive conclusions regarding the role of selection in driving phenotypic differentiation. This fact has largely been ignored in previous studies, in which Qst-based approaches were used to explore craniofacial divergence [12–14,35]. In these studies, *h*^*2*^ was assumed to be 0.55 and *c* was implicitly assumed to be 1, resulting in *c/h*^*2*^ to be greater than 1. While this might be true for some traits (for example, see skin pigmentation in Table 3), this is an anticonservative approach in our opinion, because it assumes that the heritability across populations is higher than the heritability within populations. If anything, the opposite case (*c/h*^*2*^ < 1) is more plausible as we expect environmental variation to be large across geographically distant populations. Keeping this in mind, we evaluated the sensitivity of the Qst–Fst results to the case where *c*/*h*^*2*^ < 1 [32], by determining the ‘critical value’ of *c/h*^*2*^ at which the 95% lower bound of Qst meets the 95% upper bound of Fst (Fig 4). Smaller critical values imply that phenotype-based Qst–Fst comparisons are robust to *c/h*^*2*^ assumptions. Such ‘sensitivity analyses’, while common in the molecular ecology literature, are underrepresented in human genetic and anthropological studies. Fig 4 shows the sensitivity curves for height, skin pigmentation, and nose shape traits. Skin pigmentation, which shows a very high Qst value, has a critical value of 0.25, which is much lower than 1 (Fig 4 and Table 2). The critical value of nares width is 0.50 and that of alar base width is 0.55, which are also much lower than 1 (Fig 4 and Table 2). Thus, we expect that these phenotypes would exhibit signals of accelerated divergence, even if the true values of *c/h*^*2*^ were much less than 1.

**Table 3:** Heritability of phenotypes within- and between-populations.

Phenotype	Sample size (for *h*_*g*_^*2*^)–Europeans^f^	*h*_*g*_^*2*^ (S.E)	Sample size (for *h*_*y*_^*2*^)–Admixed European and W. African^g^	*h*_*y*_^*2*^ (S.E)
Height	1,825	0.394 (0.174)	409	0.224 (0.091)
Melanin Index	1,231	0.191 (0.263)	NA	NA
Nares width	1,718	0.504 (0.187)	409	0.226 (0.094)
Alar base width	1,718	0.481 (0.188)	409	0.212 (0.093)
Nasal height	1,718	0.441 (0.186)	409	0.030 (0.076)
Nasal ridge length	1,718	0.524 (0.188)	409	0.059 (0.078)
Nasal tip protrusion	1,718	0.401 (0.191)	409	0.177 (0.088)
External surface area	1,718	0.449 (0.187)	409	0.121 (0.086)
Nostril area	1,718	0.657 (0.187)	409	0.059 (0.088)

f. *h*_g_^2^ was estimated in individuals with European ancestry.

g. *h*_*y*_^*2*^ was estimated in individuals with mixed West African and European ancestry.

### Investigating heritability of nose shape within- and among-populations

In addition to the sensitivity analysis, we wanted to demonstrate that differences in nose shape among individuals both within- and between-populations are heritable. Within-population heritability (*h*^2^) for a phenotype is traditionally estimated from data collected on large sets of twins or from pedigrees where the genetic relationships among individuals are known [36]. Recently, Yang and colleagues (2010) introduced a linear mixed model approach, which can be used to estimate an alternative statistic in unrelated individuals: the proportion of phenotypic variance explained by genotyped SNPs (*h*_*g*_^*2*^) [18]. A major advantage of this approach over traditional twin- and pedigree-based approaches is that it is applicable to data from unrelated individuals, which are more accessible and easier to collect in large numbers. We calculated *h*_*g*_^*2*^ as an estimate of *h*^*2*^ for height, melanin index, and nose shape traits using 118,420 autosomal SNPs in a sample of unrelated persons of European ancestry, implemented in the GCTA software [37] (Methods). The values of *h*_*g*_^*2*^ are provided in Table 3. The heritability for height in Europeans (*h*_*g*_^*2*^ = 0.394; N = 1,825) is similar to previously reported estimates [18]. Interestingly, the heritability of skin pigmentation is very low in Europeans (*h*_*g*_^*2*^ = 0.191; N = 1,231), which we think can be explained by reduced genetic variation at skin pigmentation loci due to strong positive selection for lighter skin in Europeans [17,21,38]. Almost all aspects of nose shape seem to be highly heritable in Europeans (*h*_*g*_^*2*^ range: 0.401–0.657 N = 1,718) (Table 3).

Estimation of the among-population heritability (*c*) is more difficult, since genetic effects between geographically distant populations can be confounded with large environmental effects. This parameter can often only be reliably estimated through ‘common garden’ experiments, in which systematic differences in the environment, which might be confounded with genetic differences, can be taken into account. However, as mentioned earlier, this approach is only possible in model organisms, which are amenable to experimental manipulation. In humans and other non-model organisms, this is a severe limitation, which can be overcome by studying naturally occurring admixed populations.

**Fig 5:**
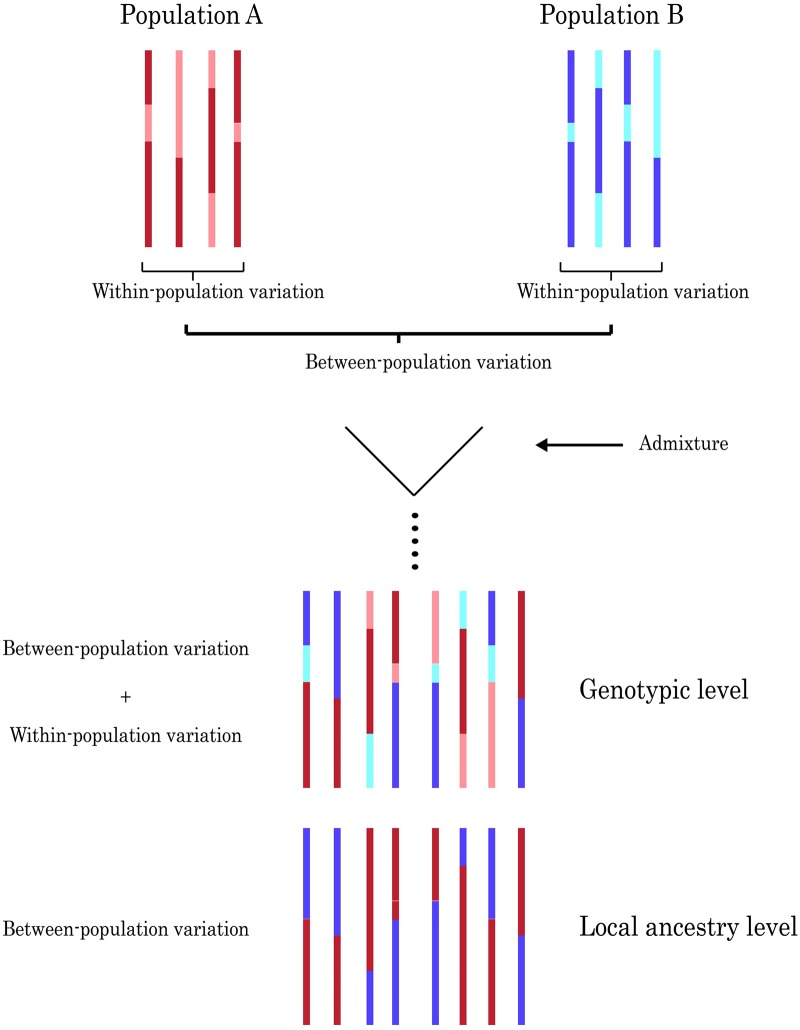
Admixture brings together within- and between-population variation in admixed individuals. Genetic variation between the two parental populations is represented by difference in color of the chromosomes, whereas genetic variation within the populations is represented by hue intensity. Admixture brings together genetic variation from both populations. On a genotypic level, genetic variation within admixed populations is composed of both within-population and between-population variation. The variation at the level of ancestry is variation between the two parental populations.

The process of admixture reunites gene pools from two or more populations, which might have diverged due to genetic drift, mutation, and selection. Admixture, which may occur repeatedly over several generations, followed by recombination, leads to chromosomes that are essentially mosaics of ancestry segments (Fig 5) [39]. In a randomly mating admixed population, ancestry segments segregate randomly with respect to the environment, which decouples the between-population genetic effects on the phenotype from the environmental effects, allowing for the estimation of the genetic variance underlying a phenotype. We propose that a method recently developed to estimate heritability in admixed populations (Zaitlen *et al*. (2014)) [40], might provide a valid estimate of *c*. Zaitlen *et al*. (2014) extend the method developed by Yang *et al*. (2010) [18], using local ancestry at SNPs, instead of genotypes, to construct the genetic relationship matrix among admixed individuals. The proportion of phenotypic variance in an admixed population that can be explained by local ancestry (*h*_*y*_^*2*^) is conceptually equivalent to *c* between the parental populations. Our reasoning is that, on a genotypic level, the genetic variation in an admixed population should be the sum of the genetic variation within the parental populations and the genetic variation between them. Variation at the scale of local ancestry only represents genetic variation between populations (Fig 5). Thus, the proportion of phenotypic variation that can be explained by local ancestry (*h*_*y*_^*2*^), should be equivalent to *c*.

Following this reasoning, we estimated *h*_*y*_^*2*^ from local ancestry at 623,625 autosomal SNPs in a sample of 409 Cape Verdeans, who derive their ancestry primarily from W. Africans and Europeans (see Methods for details and Table 3 for results). Table 3 shows that the phenotypic variation between W. Africans and Europeans in height (*h*_*y*_^*2*^ = 0.224, N = 409) is quite heritable. Results for skin pigmentation data are not presented here as they were unavailable. Differences in many aspects of nose shape are also heritable (Nares width: *h*_*y*_^*2*^ = 0.226, Alar base width: *h*_*y*_^*2*^ = 0.212, Nasal tip protrusion: 0.177, External surface area: *h*_*y*_^*2*^ = 0.121, N = 409). Other aspects of nose shape may not be as heritable between these two populations (Nasal height: *h*_*y*_^*2*^ = 0.03, *h*_*y*_^*2*^ = 0.059, N = 409). Another interesting observation from Table 3 is that estimates of *h*_*y*_^*2*^ are generally lower than estimates of *h*_*g*_^*2*^ for all traits. This suggests that for most human traits, the additive genetic variance between populations might be less than the additive genetic variance within populations, which agrees with the fact that most of the genetic variation in humans exists within populations. However, we are cautious of over-stating this conclusion since the heritabilities were estimated in individuals with W. African and European ancestry only and do not reflect the variation within and across other populations. Altogether, our results show that genetic differences underlie the variation in many aspects of nose shape, both within- and between-populations.

### Testing for adaptation to climate

**Fig 6:**
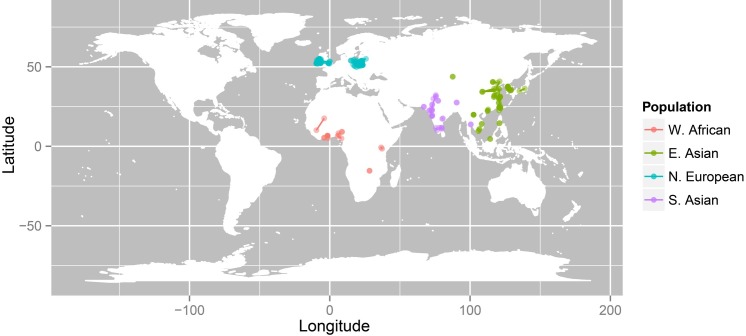
Geographic distribution of parents’ birthplaces. Individual points represent the birth locations of the parents with a line connecting two parents. A single point indicates that the two parents were born in the same location. Climate values at these locations were used to test for signals of climate adaptation.

Previously, several studies have shown that the shapes of the nasal aperture and nasal cavity are correlated with climate variables related to temperature and humidity such that individuals from cold-dry climates exhibit narrower nasal cavities compared to individuals from warm-humid climates [41,42]. We were interested in testing whether aspects of external nose shape showing unusually high differentiation across populations based on Qst–Fst analysis, show correlations with climate. For this purpose, we selected, from the subset used in the Qst analysis, females with genetic data whose parents were born in a region that coincided with their continental ancestry (N = 140) (Fig 6). This was done to assign to each individual, a climate value that was most similar to their ‘ancestral’ climate. Since we did not have genotype data available for males of Northern European ancestry, we only used females for this analysis. The genotype data were necessary to correct for genetic structure (see Methods). We tested the correlation of nares width and alar base width with three climate variables: i) mean annual temperature (hereafter referred to as temperature), ii) relative humidity, and iii) absolute humidity. The choice of these climate variables follows from the functional importance of the nose in warming and humidifying inspired air. We also tested whether skin pigmentation is correlated with UVB levels. This was used as proof of principle, since multiple lines of evidence suggest that differences in skin pigmentation across populations have evolved primarily in response to ultraviolet radiation [17,20–22]. The association between phenotypes and climate variables was tested using linear mixed models, which correct for age, BMI, and genetic similarity (Methods). Sex was not included as a covariate as only females were used. We used a likelihood ratio test (LRT) to evaluate the statistical significance of the slope between phenotype and climate variable. The LRT statistic and its corresponding p-values were generated by comparing full (climate predictor included) and reduced (climate predictor removed) models. Results are presented in Table 4.

**Table 4:** Results of test for climate adaptation.

**Skin pigmentation**^**m**^ **(N = 126)**					
** **	**Slope**^**h**^	**SE**^**i**^	**T**^**j**^	**LRT**^**k**^	**P-value**^**l**^
**UVB**	3.16E-03	5.09E-04	6.23	55.20	1.09E-13
**Nares width (N = 140)**					
	**Slope**	**SE**	**T**	**LRT**	**P-value**
**Temp**	0.107	0.035	3.09	9.24	2.37E-03
**Relative humidity**	-3.626	2.192	-1.65	2.71	0.100
** Absolute humidity**	0.149	0.054	2.73	7.28	6.97E-03
**Alar base width (N = 140)**					
** **	**Slope**	**SE**	**T**	**LRT**	**P-value**
**Temp**	0.065	0.033	1.97	3.82	0.051
**Relative humidity**	-1.642	2.083	-0.79	0.62	0.431
**Absolute humidity**	0.109	0.052	2.09	4.30	0.038

h. slope between phenotype and climate variable.

i. standard error of the slope.

j. t-statistic for the slope.

k. likelihood ratio between full and reduced model.

l. p-value for the likelihood ratio test.

m. In the case of skin pigmentation, we used melanin index to generate the slope but the inverse of melanin index to generate the LRT and P-value.

As expected, we see a strong relationship between skin pigmentation and UVB (N = 126, T = 6.23, LRT = 55.20, p-value = 1.09E-13). The positive values of the slope and t-statistic indicate that people tend to be darker in regions with higher exposure to UVB and vice versa. This pattern provides further support to the notion that adaptation to ultraviolet radiation has had an important role in the evolution of skin pigmentation in humans. Nares width is significantly correlated with temperature (N = 140, T = 3.09, LRT = 9.24, p-value = 2.37E-03) and absolute humidity (N = 140, T = 2.73, LRT = 7.28, p-value = 6.97E-03). The positive slopes indicate that individuals from warm-humid climates, on average, tend to have wider nares whereas individuals from cool-dry climates tend to have narrower nares. This is consistent with previously reported patterns of ecogeographic variation in the width of the nasal aperture and nasal cavity [41,42]. Nares width was not significantly correlated with relative humidity (N = 140, T = -1.65, LRT = 2.71, p-value = 0.100). This is not surprising since absolute humidity is more important for the physiological functioning of the nose than relative humidity [43]. Alar base width is neither significantly correlated with temperature (N = 140, T = 1.97, LRT = 3.82, p-value = 0.051) nor with relative humidity (N = 140, T = -0.79, LRT = 0.62, p-value = 0.431), and is only mildly correlated with absolute humidity (N = 140, T = 2.09, LRT = 4.30, p-value = 0.038). This might suggest that it is not the width of the nose *per se* but the width of the nares that is under selection pressure. However, even nares width is not as strongly correlated with temperature and humidity as skin pigmentation is to UVB, suggesting that the strength of selection exerted by temperature on nose width is likely to be much weaker than the selection pressure imposed by UVB exposure on skin pigmentation.

**Fig 7:**
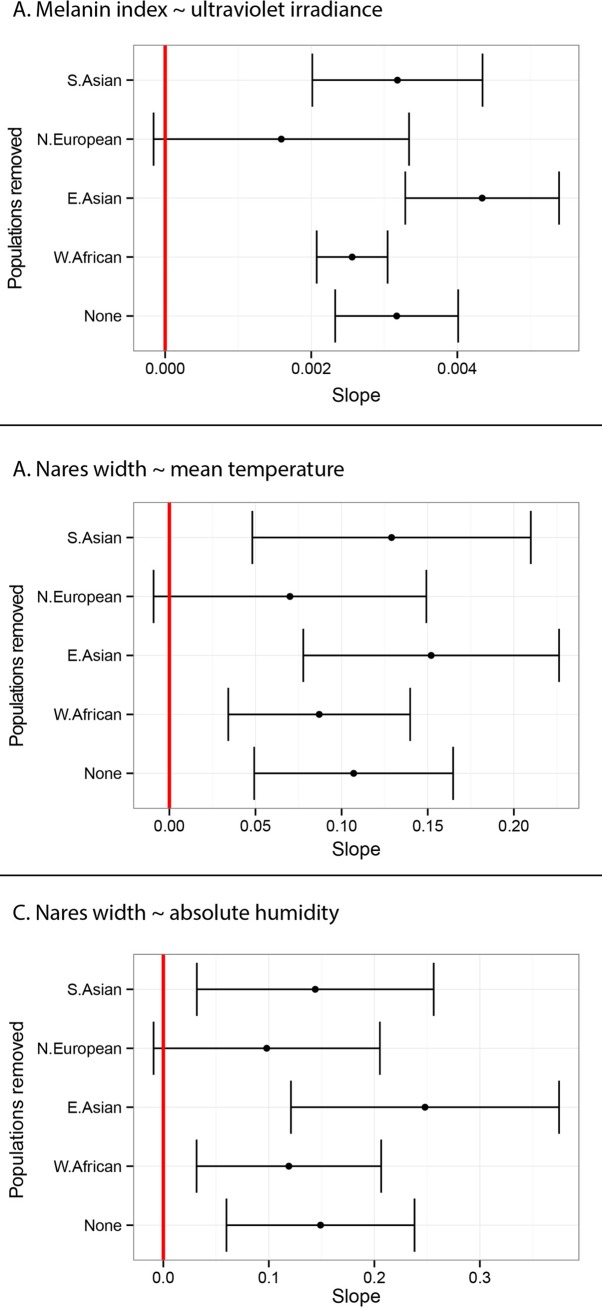
The effect of removing each population on phenotype-climate correlations. The point estimate and the 95% confidence intervals of the phenotype-climate slopes after removing each population (on the y-axis) are shown for A) skin pigmentation and UVB, B) nares width and temperature, and C) nares width and absolute humidity. In all cases, the 95% confidence interval of the slope overlaps with zero (red vertical line), only when the Europeans are removed.

Following a suggestion by one of the reviewers, we investigated whether the observed phenotype-climate correlations were driven by any one population. To do so, we re-estimated the phenotype-climate slopes (skin pigmentation vs UVB and nares width vs temperature) after removing each population in turn. Fig 7 shows that the 95% confidence intervals of the slope overlap with zero (red line in Fig 7) only when the N. Europeans are removed, suggesting that both phenotype-climate correlations are driven primarily by the inclusion of N. Europeans. This effect might be indicative of positive selection for lighter skin pigmentation and narrower nares in higher latitude populations. However, because our sampling of populations at higher latitude is limited (Fig 6), this result requires further investigation.

## Discussion

The diversity of facial features across human populations has fascinated scientists for a long time. Even though genetic drift has played a predominant role in human evolution, external physical traits such as facial shape and skin pigmentation, because of their proximity to the environment, have also likely been influenced by natural selection. Substantial evidence has accumulated supporting the hypothesis that differences in skin pigmentation across human populations have evolved largely in response to selection pressures imposed by exposure to ultraviolet radiation [20]. How selection may have affected facial shape, a trait that is also quite variable between populations, is unclear, likely because it has received much less attention to date. Given the complexity of the face, we have chosen to study one particularly interesting and variable part of the face; the nose. The broad question driving this study is: Has climate adaptation played an important role in influencing variation in human nose shape? To answer this question, we formulated two hypotheses: i. divergent selection has been involved in the differentiation of certain aspects of nose shape across populations, and ii. climate is the agent of selection in cases where divergent selection can be invoked.

To test the first hypothesis, we used Qst–Fst comparisons to investigate whether the mean difference in nose shape among populations is greater than that expected under genetic drift alone. Estimation of Qst relies on stipulating the within- and among-population components of phenotypic variance that are due to additive genetic effects. These variance components are ideally calculated through experiments in which the effects of environmental variables can be controlled [28]. This poses a practical and ethical challenge in non-model organisms, such as humans, who are not amenable to the type of experimentation required. To circumvent these limitations, studies often calculate Qst directly from phenotype data under the assumption that the within-population heritability (*h*^*2*^) is equal to the among-population heritability (*c*). Qst calculated this way is probably best considered to be the ‘minimum’ Qst proposed by Relethford (1994) [35]. However, the notion that this takes the minimum value of Qst relies on *h*^2^ always being less than *c*, which is anticonservative as has been noted previously [32]. In fact, genetic variation between-populations is likely to be less than the genetic variation within-populations for most phenotypes. Our approach is similar to previous studies in that we also estimated Qst assuming *c* = *h*^*2*^. However, we used sensitivity curves to evaluate the behavior of Qst to cases where *c* < *h*^*2*^. In addition, we demonstrate that both within- and between-population variation in nose shape are heritable.

We carried out Qst–Fst comparisons for seven nose shape traits: nares width, alar base width, nasal height, length of the nasal ridge, nasal tip protrusion, external surface area, and nostril area. We found that the divergence in the width of the nares and alar base deviate from neutral expectations, and that these results are robust to the *c* = *h*^*2*^ assumption. This suggests that the width of the nares and alar base may have evolved across populations due to divergent selection.

Next, we hypothesized that the divergence of these two traits is driven by climate adaptation. To investigate this, we tested whether the spatial distribution of these traits is correlated with mean annual temperature and humidity. Our results show that nares width is strongly correlated with temperature and absolutely humidity. The positive direction of the effects indicate that wider noses are more common in warm-humid climates, while narrower noses are more common in cold-dry climates. Nares width is not, however, correlated with relative humidity. This is not surprising since absolute humidity levels are likely more important for respiration than relative humidity [43]. Alar base width is only weakly, if at all, correlated with temperature and absolute humidity, suggesting that the signal of climate adaptation might be specific to the width of the nares. Computational fluid dynamics (CFD) studies show that the geometry of the nose is important for its respiratory functions [5,44]. Inhaled air reaches 90% of the required temperature and humidity levels before even reaching the nasopharynx, implicating the nasal cavity, especially the turbinates, as the major conditioning apparatus in the respiratory tract [4,5]. We also know that the geometry of the nasal airways influences the velocity of inspired air [4,7,45]. Narrow airways in cold-dry climates might allow better conditioning by increasing the turbulence in inspired air as it reaches the turbinates, thereby facilitating contact with the nasal mucosa [5]. However, we note that nostril area does not show unusually high differentiation across populations, which suggests that it is not the size of the nostrils but the shape that might be functionally important.

Another hypothesis regarding the function of the nose that has received less attention compared to the air-conditioning hypothesis is that of selective brain cooling (SBC) [46]. Some large mammals are known to regulate brain temperatures in hyperthermic conditions, often caused by sustained exercise [47,48]. This ability is mostly reported in mammals with a carotid rete, which is a network of capillaries in a cavity under the brain called the cavernous sinus. Arterial blood leaving the rete eventually perfuses the brain through the Circle of Willis. Venous blood traveling back from the nasal mucosa interacts with the carotid rete in the cavernous sinus and is thought to cool the blood entering the brain in arid-zone mammals, such as Oryx [49], as well as in winter-acclimatized animals, such as reindeer [50]. Although, humans do not possess such a rete and instead have a single artery going through the cavernous sinus, which has led some to question the role of SBC in humans [51], proponents argue that the carotid rete is not a pre-requisite as SBC is present in some animals who don’t possess a rete, such as horses [52]. Since SBC in humans is highly debated, the role of nose shape differences in contributing to differences to this physiological process, while worth mentioning here, is highly speculative.

Climate may not have been the only factor in contributing to nose shape differences across populations. In fact, we show that temperature is only weakly correlated with nares width, especially when compared with the correlation between skin pigmentation and UVB. What then could be the selective agent driving the divergence of nose shape? We mentioned earlier that all aspects of nose shape studied here are sexually dimorphic, which raises a number of questions. Why does this sexual dimorphism exist? Is it merely a by-product of circulating hormones leading to differences in growth and development in early adulthood, or does it have an adaptive function, such as signaling sex to other males and females? Sexual selection has likely played an important role in human evolution, as evidenced by the presence of sexual dimorphism in many physical traits (e.g., height, waist-hip ratio, facial hair, and breasts to name a few). Could cultural differences in perceptions of dominance and attractiveness have had a role in the divergence of nose shape [53]? Could these perceptions have arisen to select mates who signal adaptation to the local environment? Indeed, ecological selection and sexual selection could reinforce each other, accelerating phenotypic divergence across populations in spite of continued gene flow [54]. These are interesting avenues of research, which need to be considered in order to sketch out a more complete picture of the evolution of the human nose.

The investigation of nose shape evolution with respect to climate adaptation, while interesting anthropologically, is also relevant medically. As humans are becoming more of a global community, the study of local adaptation is becoming more important to understanding health risks involved in living in ‘foreign’ climates. Obvious examples of such health risks are of increased risk of sunburn, skin cancer, and folate deficiency in light-skinned individuals exposed to high UVB, and of low birth weight and chronic mountain sickness associated with hypoxia at high altitudes [20,55]. Does the morphology of the external nose, or that of the inner nasal cavity affect risk of respiratory disease in different climates? It’s difficult to say at this point. While our findings provide support for the idea that differences in aspects of nose shape may have evolved across populations as a result of climate-related selection pressures, something that has been demonstrated previously using craniometric data [2,12,13,41,42], we note that the signal of climate adaptation is not very strong, especially when compared to skin pigmentation. This could be due to weaker selection pressure or selection on standing variation, but also due the sparse sampling of populations shown here, which is a limitation of this study. These results will need to be replicated in a larger set of populations. We expect that studies incorporating diverse populations who have been living long-term in a range of environments, such as the tropics, deserts, and circumpolar regions, will nicely fill in the gaps. Especially useful would be representation of populations from higher altitude regions, such as Andeans, Tibetans, and Ethiopians, who not only have to cope with the stress of a cold and dry climate, but also that of low atmospheric oxygen levels [56]. It would also be informative to study non-human primates in this context, who occupy a variety of climes and exhibit extensive variation in nose morphology. Finally, future studies should also focus on genome-wide association studies (GWASs) to identify variants contributing to nose shape. With increasing interest in identifying loci associated with facial shape, some GWASs have recently identified a number of nose shape loci [57–60]. Genetic variation at these loci will be informative about the nature of selection, as well as for inferring the timing of selection events.

## Materials and methods

### Ethics statement

Data collection was carried out with informed consent of participants and with approval from the institutional review boards (IRBs) at The Pennsylvania State University (IRB# 32341, IRB# 45727, IRB# 44929) and at The University of Illinois Urbana-Champaign (IRB# 13103). All participants provided written informed consent.

### Participant recruitment and description of the data

The data used in this paper are part of a larger dataset that were collected in various locations through studies based at the Pennsylvania State University and at the University of Illinois at Urbana-Champaign. The majority of these data were collected in the United States at the Pennsylvania State University, at the World Science Festival in New York, and at the University of Illinois at Urbana-Champaign. Data collection in Europe took place in Dublin (Ireland), in Rome (Italy), in Warsaw (Poland), and in Porto (Portugal). The Cape Verdean data were collected in collaboration with Dr. Sandra Beleza as described in Beleza *et al*. [61]. All data were collected through informed consent from the participants with approval from the institutional review boards at The Pennsylvania State University, at the University of Illinois at Urbana-Champaign, and the National Ethical Committee for Health Research of Cape Verde.

The phenotype data collected include three dimensional (3D) images, skin pigmentation, standing height, and body weight. 3D images of participants were taken using the 3dMD system (*3dMD System*, *Atlanta*, *GA*). Skin pigmentation was measured with melanin index using Derma Spectrometer (*Cortex Technology*, *Hadsund*, *Denmark*). Melanin index is defined as the 100 x log_10_ (1/percentage reflectance at 650 nM) [17]. We took three readings of melanin index from the inner left and right arm and used the mean of the six measurements for each individual. Statistical testing was performed using the inverse of melanin index since it is more normally distributed (S7 Fig) but the use of untransformed melanin index produces similar results and does not affect our conclusions. Demographic information was collected through questionnaires and included self-reported ancestry and sex, participant’s birthplace, the birthplaces of the parents and grandparents, and the locations where the participant was raised.

The total number of participants was 4,257. Of these, we selected 2,637 individuals based on genetic ancestry and availability of phenotypic and covariate data, as described in the following sections. A summary of the sample sizes used in different analyses is provided in Table 5. For a more detailed and accurate representation, refer to the Excel spreadsheet in the supplementary materials (S1 File), which provides individual-level data for all samples used to generate the results presented here.

**Table 5:** Sample sizes by ancestry group and sex used in each analysis.

Analysis/ Group	W.African		S.Asian		E.Asian		N.European		‘Other’ European		Cape Verdean		Total
	F^n^	M	F	M	F	M	F	M	F	M	F	M	
Qst–Fst	30	10	31	42	84	43	145	91					476
Climate adaptation	10	0	17	0	41	0	74	0					140
Estimation of *h*_*g*_^*2*^							73	0	1,138	614			1,825
Estimation of *c*											245	164	409
Total	30	10	31	42	84	43	145	91	1,138	614	245	164	2,637

n. F = female, M = male.

### Estimation of genetic ancestry

#### Genotyping and quality control

Out of the 4,257 participants, we had genotype data for 3,746 individuals. Since the data were collected as part of different studies over a long period of time, the samples were genotyped on four different genotyping arrays, namely the Illumina HumanHap300v1 BeadChip, the Illumina Infinium HD Human 1M-Duo Beadarray (*Illumina*, *Inc*., *San Diego*, *CA*), and the 23andMe v3 and v4 arrays (*23andMe Mountainview*, *CA)*. In order to select individuals based on genetic ancestry, we needed to merge genotypes across platforms into a single database. We took several steps to ensure there were no batch effects due to genotyping platform, which could potentially lead to biases in ancestry estimation and Fst calculation. First, we removed SNPs that were not present on all four platforms to ensure there were no systematic differences due to missingness. Second, we performed quality-control separately for each platform by removing individuals and SNPs with a genotyping rate of less than 90%. Finally, we removed palindromic (AT/GC) SNPs and recoded the data in the Illumina A/B format [62] to ensure strand differences across platforms were not an issue. This sufficiently removes any potential batch effects due to platform as evidenced by the observation that in a multidimensional scaling plot, individuals cluster by ancestry and not by platform (S1 Fig). At this stage, the genotypes were merged across platforms into a single dataset. We further removed non-autosomal SNPs from the merged dataset and pruned for linkage disequilibrium (using a pairwise r^2^ cutoff of 0.5). This resulted in 118,420 autosomal SNPs. Related individuals were identified using identity-by-state (IBS) analysis (IBS > 0.8). Individuals were removed to minimize relatedness and maximize sample size (e.g. In the case of a mother-father-siblings quartet, the siblings were removed). These QC steps were carried out in Plink 1.9 [63,64].

#### Genomic ancestry estimation and principal components analysis

We estimated genetic ancestry for all individuals with available genotype data (N = 3,746) using an unsupervised clustering approach in ADMIXTURE. To assist with the clustering and interpretation of the resulting clusters, we merged this dataset with genotypes from the HapMap dataset for the same SNPs (Populations included: YRI, LWK, CEU, TSI, CHB, CHD, JPT, GIH, MEX; N = 988). We ran ADMIXTURE for three values of k (k = 5, 6, 7), and visually determined the optimum clustering scheme to be k = 6 for our purposes. To further visualize fine-scale structure, we carried out unsupervised principal component analysis (PCA) on the genotype data in Plink 1.9 [64]. A combination of filters based on the ADMIXTURE output and the PCA scores allowed us to select individuals from specific populations of interest. These are described below separately for each analysis.

### Qst–Fst analyses

#### Sub-selection of individuals

For the Qst–Fst analyses, we were interested in selecting individuals with ancestry primarily from one of four populations: i) North European, ii) West African, iii) East Asian, and iv) South Asian. Much of this was based on genetic ancestry calculated from dense genotype data. However, for some individuals (see *Northern European* below), self-reported ancestry information, corroborated through the birthplace of all four grandparents, was used where genotype data were unavailable.

**Fig 8:**
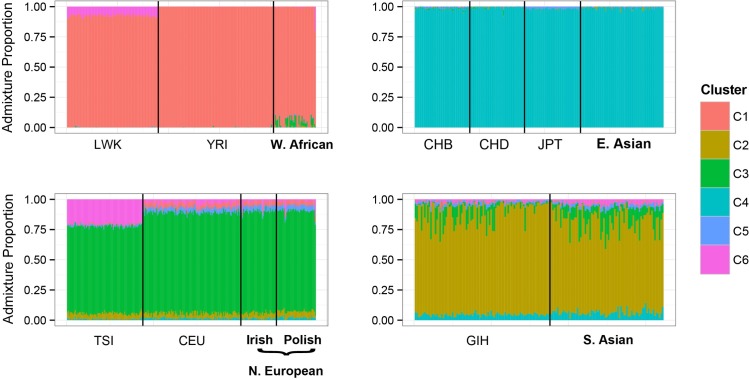
ADMIXTURE Ancestry proportions (k = 6) of genotyped individuals used in Qst–Fst analyses. Their ADMIXTURE proportions are compared with samples from the HapMap dataset. Each vertical bar in the panels is an individual and the colors represent the proportion of ancestry derived from each of 6 clusters (k = 6). In each panel, the HapMap samples are arranged on the left with a three-letter acronym for the population they are from (e.g. GIH refers to the Gujarati Indians from Houston) listed under them. The samples from our dataset are arranged to the right of the HapMap samples with their population designation (e.g. South Asian) under them.

*West African (N = 40)*: We identified 40 individuals who had close to 90% or greater ancestry from ADMIXTURE cluster 1. ADMIXTURE cluster 1 represents ancestry from West Africa, given that individuals from the HapMap YRI (Yoruba in Ibadan, Nigeria) derive most of their ancestry from ADMIXTURE cluster 1 (Fig 8). These 40 individuals were included in the ‘West African’ population group.

*East Asian (N = 127)*: Comparison with the HapMap CHB (Han Chinese in Beijing, China), HapMap CHD (Chinese in Metropolitan Denver, Colorado), and HapMap JPT (Japanese in Tokyo, Japan) samples shows that cluster 4 represents genetic ancestry from East Asia (Fig 8). We selected 166 individuals who had 90% or more ancestry from ADMIXTURE cluster 4 to be included in the ‘East Asian’ population group (Fig 8). We further examined the distribution of the PCA plots and removed six individuals who clustered far from the CHB, CHD, and JPT samples. The clustering of the remaining 160 individuals around the CHB, CHD, and JPT samples is shown in S2 Fig. Of these, we had high quality 3D images and complete covariate data for 127 individuals, who were then used in our analyses.

*South Asian (N = 73)*: 98 individuals in our dataset had more than 60% ancestry from ADMIXTURE cluster 2, which seems to represent the South Asian cluster (Fig 8). These 98 individuals were added to the ‘South Asian’ population group. While the cutoff here seems liberal compared to that used for the other populations, it is only meant to be a rough guide and was chosen by comparing with the ancestry proportions of the HapMap GIH (Gujarati Indians in Houston, Texas) samples (Fig 8). However, we examined the distribution of the 98 individuals around the GIH (Gujarati Indians in Houston, Texas) samples using PCA plots (S2 Fig), and noticed that six individuals appear to fall far from the main cluster, who were subsequently removed. Of the remaining, we had high quality 3D images and complete covariate data for 73 individuals, who were used for further analyses.

*Northern European (N = 236)*: Our full dataset had a large number of individuals with European ancestry from different regions of Europe. In order to minimize structure within Europe, we only retained individuals with Northern European ancestry. Thus, we restricted the analysis to Europeans collected in Ireland (N = 151) and Poland (N = 85) who reported that the ancestry of all four of their grandparents was from the region of sampling. More details for these samples are described in Candille *et al*. [65]. Of this set, we had genotypes for 73 females (Irish = 37, Polish = 36) but none for males. This is the reason why only females were included in the phenotype-climate analyses, where genotypes were used to construct the genetic relationship matrix. The ADMIXTURE results for these women are shown in Fig 8 alongside the results of the CEU (Utah residents with Northern and Western European ancestry from the CEPH collection) and TSI (Tuscans from Italy) samples.

#### Qst calculation

Estimation of the variance components, $\sigma_{pb}^{2}$ and $\sigma_{pw}^{2}$, was carried out using a linear model in which population group was treated as a fixed effect, with sex, age, and BMI as covariates. If *MSB* is the mean square among populations, *MSW* is the mean square within populations, *n*_*i*_ is the number of individuals in the i^th^ populations, and *a* is the number of populations, $\sigma_{pb}^{2}$ and $\sigma_{pw}^{2}$ can be estimated as described as follows [10,66,67]:

$$n_{0}=\frac{1}{a-1}(\sum_{i}^{a}n_{i}\;\text{-}\;\frac{\sum_{i}^{a}n_{i}^{2}}{\sum_{i}^{a}n_{i}})$$ (3)

$${\hat{\sigma }}_{pw}^{2}=MSW$$ (4)

$${\hat{\sigma }}_{pb}^{2}=\frac{MSB-MSW}{n_{0}}$$ (5)

#### Fst calculation

Wright’s Fst measures the genetic divergence between two or more populations. We estimated the Fst across the four populations for the LD-pruned set of 118,420 autosomal SNPs, described above, using Weir and Cockerham’s *θ* [68]. The genomic Fst distribution in shown in S3 Fig.

#### Qst–Fst comparison

We used a non-parametric bootstrap approach to test whether the Qst of a phenotype is significantly greater than the Fst. This was done by computing Qst from subsamples generated by randomly selecting individuals with replacement, within population and sex such that the number of males and females in each population remains unchanged. Values of Fst were simulated by randomly sampling from the Fst of 118,420 SNPs (S3 Fig). We generated 10,000 such pseudo-samples and, for each simulated value of Qst and Fst, computed the difference Qst–Fst. High values of Qst–Fst are indicative of divergent selection, and the empirical p-value for this test was determined by calculating the proportion of Qst–Fst values that are less than zero.

### Estimation of $h_{g}^{2}$ and *c*

#### Estimation of $h_{g}^{2}$

**Fig 9:**
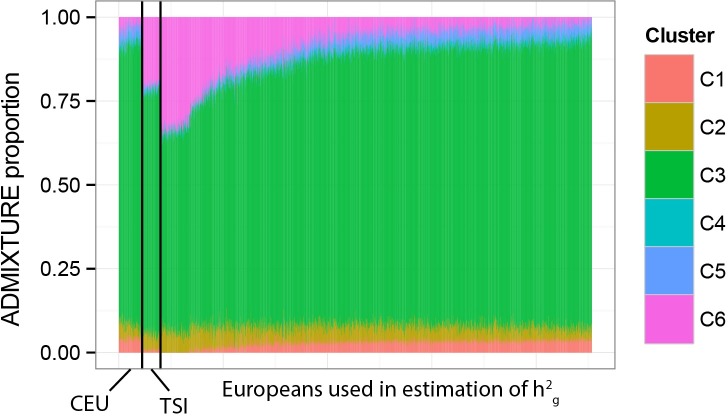
ADMIXTURE ancestry proportions (k = 6) of 1,825 individuals with primarily European ancestry, who were used to estimate *h_g_^2^*. Each bar represents an individual and the different colors represent the ADMIXTURE proportions from six clusters (k = 6). The ADMIXTURE proportions for HapMap European populations (CEU and TSI) are shown on the left for comparison.

For the calculation of $h_{g}^{2}$, we identified 1,825 unrelated individuals with primarily European ancestry, who derived more than 80% of their combined genetic ancestry from Cluster 3 and Cluster 6 based on the ADMIXTURE output (Fig 9). This cutoff was chosen based on comparison with the genetic ancestries of CEU (Utah residents with Northern and Western European ancestry from the CEPH collection) and TSI (Toscans in Italy) individuals from the HapMap dataset (Fig 9). We used GCTA to calculate the genome-wide kinship matrix for the 1,825 individuals from 118,420 autosomal SNPs, which overlapped across all four platforms. Of the 1,825 individuals with European ancestry, we had measured height for 1,825, measured melanin index for 1,231, and 3D photos for 1,718 individuals. From these data, we used GCTA to estimate the proportion of phenotypic variance explained by genotyped SNPs (*h*_*g*_^*2*^) for height, skin pigmentation, and nose shape aspects. The top four genetic PCs were included as covariates in the model to correct for population structure, which can inflate heritability estimates [18,69,70]. Also included in the model were sex, age, and BMI.

#### Estimation of c

**Fig 10:**
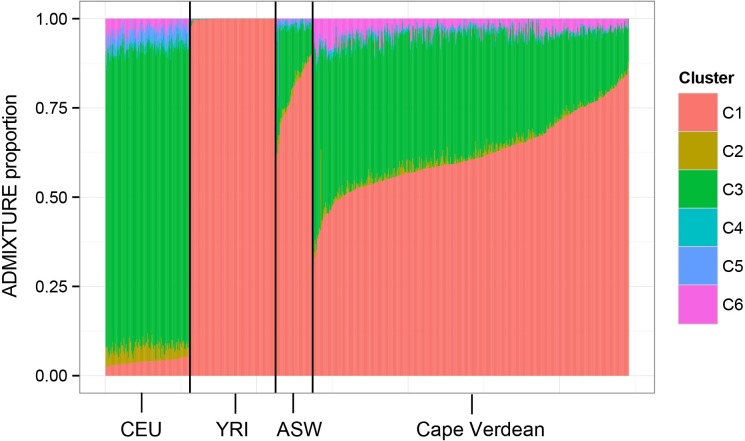
ADMIXTURE ancestry proportions (k = 6) of 409 Cape Verdeans, who were used to estimate *c*. Each bar represents an individual and the different colors represent the ADMIXTURE proportions from six clusters (k = 6).

We estimated the proportion of phenotypic variance between populations due to additive genetic effects (*c*) in an admixed population from Cape Verde, who derive their ancestry primarily from W. Africans and Europeans (Fig 10), using the approach described in Zaitlen *et al*. [40]. 697 Cape Verdeans were genotyped on the Illumina Infinium HD Human 1M-Duo Beadarray (*Illumina*, *Inc*., *San Diego*, *CA*) for a total of 1,016,423 SNPs. This was reduced to 623,625 autosomal SNPs after removing palindromic SNPs, SNPs that did not overlap with the HapMap dataset, and other SNPs based on filters described in Beleza *et al*. [17]. We had high quality 3D images and complete covariate data for 409 of the 697 Cape Verdeans who were genotyped. For these individuals, we calculated local ancestry at 623,625 autosomal SNPs with LAMP-LD [71] using phased genotypes from the HapMap CEU and YRI samples as reference. Averaging local ancestry across all SNPs yields an estimate of global ancestry that is highly correlated with estimates obtained from ADMIXTURE using k = 2 (r^2^ ∼ 1) (S4 Fig). We used GCTA to calculate a kinship matrix based on local ancestry and to estimate the proportion of phenotypic variance explained by local ancestry (*h*_*y*_^*2*^). Global ancestry, calculated from the mean of local ancestry across all SNPs, was included as a covariate to correct for ancestry stratification and any environmental variables that might covary with ancestry [16,72]. Sex, age, and BMI were also included as covariates.

### Phenotype-climate associations

#### Testing for local adaptation

The primary focus here was to answer the question: Does nose shape variation between geographically distant populations show signals of local adaptation? To answer this, we needed to ascribe a climate value to every individual that can serve as a proxy of their ancestral environment. For this reason, we selected individuals used in the Qst–Fst analyses who reported at least one parent’s birthplace. These were further filtered to retain individuals whose parents’ birthplaces coincided with their continental ancestry, for example, East Asian individuals whose parents were born in the United States were removed. Finally, we restricted the analyses to females since genetic data were not available for males sampled in Ireland and Poland. This resulted in 140 females with genetic, phenotypic, and geographic data. We have plotted the geographic distribution of the parents of these individuals in Fig 6 with a line connecting the two parents of each individual. A single dot means that the parents were born in the same location. These locations were geocoded to obtain the latitude and longitude in decimal degrees using the ggmap package in R [73,74].

Gridded climate data were obtained in latitude/longitude coordinate system. Climate grids with mean monthly values for temperature (measured in degrees Celsius), and vapor pressure (measured in hectopascal or hPa) at 0.5° resolution for the period between 1901 and 2015 were obtained from The Climate Research Unit TS v3.24 Dataset (https://crudata.uea.ac.uk/cru/data/hrg/cru_ts_3.24/) [75]. Ground-level Ultraviolet-B (UVB) irradiance data (expressed in J/m^2^), for the period between 1996 and 2005, generated by the Earth Probe Total Ozone Mapping Spectrometer (EP/TOMS) Team at 0.5° resolution, were downloaded from the Goddard Earth Sciences Data and Information Services Center (GES DISC) website (https://disc.gsfc.nasa.gov/) [76]. Relative humidity and absolute humidity grids were derived from temperature and vapor pressure as described in Maddux *et al*. (2016) [43]. For each of the four climate variables (UVB, temperature, absolute humidity, and relative humidity), a single grid was constructed by averaging across all months within the time period to represent long-term climate. R packages RnetCDF [77] and Raster [78] were used to read gridded data and to extract climate values at each location. We used climate values at the parents’ birthplace to represent the ancestral climate for each individual. Where the birthplace of both parents was known, the mean climate values extracted at the two locations was used. Both parents usually come from nearby regions, at least on a continental scale (Fig 6), and the climate values for the parents are quite similar (S5 Fig).

We used a linear mixed model to test the relationship between phenotype and climate variables, while correcting for age and BMI. We took non-independence among observations due to genetic similarity into account by allowing the observations to be correlated according to the SNP-based genome-wide kinship matrix. In matrix notation, the linear model takes the following form:

Y=Xβ+ε (6)

Where *β* are fixed effects and *ε* is the random error term. Thus, *ε* ∼ *N*(0,*Gσ*_*e*_), where *G* is the genome-wide kinship matrix, which was constructed from the genotypes at 118,420 SNPs in GCTA [37]. The slopes were estimated using maximum likelihood, implemented in the spaMM package in R [74,79]. P-values for each slope were generated from the likelihood ratio between full and reduced models (fit by removing each climate predictor).

### Morphometric measurements

#### Imaging processing

We captured high resolution 3D images of participants’ faces using the 3dMD Face system (*3dMD Atlanta*, *GA*). Five positioning landmarks (two on the inner corner of the eyes, two on the outer corners of the mouth, and one on the tip of the nose) were placed on each face in order to establish facial orientation. A spatially dense mesh of 7,150 quasi-landmarks (QLs) was mapped onto each image and its reflection. As a result of this mapping, the vertices and triangles are homologous across individuals [80]. Generalized Procrustes Superimposition [24] was carried out on both sets of images (original and reflected) to remove differences in position and orientation. The Procrustes coordinates of the original and reflected image for each participant were then averaged to remove effects of bilateral asymmetry. We selected the nose region (see outline in Fig 1), which is comprised of 709 out of the 7,150 QLs making up the entire face, for further downstream analyses.

#### Linear distances

Linear distances were calculated using three mid-line landmarks; i. nasion (n), ii. pronasale (prn), iii. subnasale (sn), and two sets of paired landmarks; iv. left and right alar curvature (al_l_ and al_r_), and v. left and right alar base (ac_l_ and ac_r_). These landmarks have been defined previously [1,26] and their approximate positions are shown in Fig 1. Two different observers placed each of these seven landmarks three different times on the spatially-dense QL template mesh that was mapped on all the faces. Because the QLs are homologous across individuals, we can automatically select corresponding landmarks for all individuals by indicating these on the QL template. This is possible because any point on the template surface can be expressed as a function of QLs using barycentric coordinates on triangles. Using this approach, we obtained the x, y, and z coordinates of the above-mentioned landmarks for each individual. The Euclidean distances (in mm) were then calculated separately for each of the six replicate placements, which were averaged across replicates and then averaged between the two observers, to obtain mean distances for each individual.

#### Nostril area

To measure area of the nostrils, the observers defined regions around the nostrils (approximate location shown in Fig 1) three different times. The area of a region was calculated as the total area of the polygons within the region. Using this approach, we calculated the mean area of the right and left nostrils (in mm^2^) for each replicate. This was then averaged across replicates for the same observer, then across observers to obtain the mean nostril area for each individual.

#### External surface area

The external surface area of the nose (also in mm^2^) was calculated by summing the areas of polygons bounded by the 709 QLs, defining the entire nose region (outlined in Fig 1).

#### Intra-observer error and inter-observer reliability

We calculated the standard deviation in the measurement of linear distances (in mm) and nostril area (in mm^2^) made by the two observers to estimate the degree of intra-observer error. The quantiles for these standard deviations are shown in Table 6. The measurements from Observer2 are slightly more internally consistent than Observer1. For Observer1, nasal tip protrusion was the least precise distance measure with 95% of the standard deviations falling between 0.791mm and 1.165mm, with error for the remaining distances well under 1mm. The error distribution for nostril area was similar for both Observer1 (95% CI: 0.914–1.930) and Observer2 (95% CI: 0.888–2.243).

**Table 6:** Intra-observer error and inter-observer reliability.

Variable	Intra-observer error°				Inter-observer reliability
	Observer1		Observer2		Intraclass correlation coefficient (ICC)
	Lower– 2.5%	Upper– 97.5%	Lower– 2.5%	Upper -97.5%	
Nasal width	0.140	0.545	0.014	0.058	0.994
Alar base width	0.009	0.227	0.014	0.198	0.998
Nasal height	0.226	0.495	0.067	0.146	0.991
Nasal ridge length	0.329	0.605	0.063	0.096	0.984
Nasal tip protrusion	0.791	1.165	0.058	0.123	0.819
Nostril area	0.914	1.930	0.888	2.243	0.979

o. Intra-observer error measured in mm for distances and mm^2^ for nostril area.

Inter-observer reliability was evaluated using intraclass correlation coefficient (ICC). ICC for each measure was calculated using linear mixed models where the observer was treated as fixed effect and subject was treated as random effect. This was carried out using the nlme package in R [74,81]. With the exception of nasal tip protrusion (ICC = 0.819), the ICCs for all other measures were quite high (ICC > 0.95). This concordance is evident by comparing the mean measurements between Observer1 and Observer2 (S6 Fig)

## Supporting information

S1 Fig **MDS plots (k = 2) showing clustering of genotyped individuals by A) genotyping platform and B) by ancestry.** In B) the genotyped individuals from the study set are shown in black, while the HapMap samples are shown in color for reference. The plot shows that individuals cluster by ancestry and not by genotyping platform. Thus, batch effects due to genotyping platform are unlikely to lead to artifacts in ancestry inference.
(TIF)

S2 Fig **PCA plots showing clustering of genotyped individuals by A) South Asian and B) East Asian ancestry around HapMap reference samples.** The colored points are reference samples from the HapMap dataset and the grey points are individuals from our dataset. A) South Asian individuals are shown with the HapMap GIH samples and B) East Asian individuals are shown with the combined East Asian sample (CHB+JPT+CHD) from the HapMap dataset. The red lines indicate the cutoff for removing individuals who appear to cluster far away from the main cluster. The individuals who were removed, based on clustering, are shown as triangles, whereas all other individuals are shown as circles.
(TIF)

S3 Fig The genome-wide Fst distribution calculated using autosomal markers. The distribution was generated from 118,420 autosomal SNPs using Weir and Cockerham’s *θ*.
(TIF)

S4 Fig Global ancestry calculated using LAMP-LD is highly correlated with global ancestry calculated using ADMIXTURE. ADMIXTURE-based ancestry estimates were calculated using a k = 2 assumption. LAMP-LD-based ancestry estimates were calculating the mean local ancestry across 623,625 autosomal SNPs.
(TIF)

S5 Fig The climate values at parents’ birth locations are highly correlated. A) Mean annual temperature is measured in degrees Celsius. B) Annual aridity index is a ratio. C) UVB irradiance is measured in J/m^2^.
(TIF)

S6 Fig Mean measurements made by Observer1 and Observer2 are highly correlated with each other. (TIF)

S7 Fig **QQplots showing that the residuals for A) the inverse of melanin index are more normally distributed than B) untransformed melanin index.** The points are residuals from linear models where population, sex, age, and BMI were used as predictors.
(TIF)

S1 Table Analysis of variance results for height, skin pigmentation, and nose shape measurements. (DOCX)

S1 File Excel spreadsheet containing de-identified data used in the analyses. (ZIP)

## References

[1] Farkas LG, Kolar JC, Munro IR. Geography of the nose: a morphometric study. Aesthetic Plast Surg. 1986;10: 191–223. 3812136 10.1007/BF01575292

[2] Franciscus RG, Long JC. Variation in human nasal height and breadth. Am J Phys Anthropol. 1991;85: 419–427. 10.1002/ajpa.1330850406 1928315

[3] Negus VE. Introduction to the comparative anatomy of the nose and paranasal sinuses. Ann R Coll Surg Engl. 1954;15: 141–171. 13198060 PMC2377799

[4] Hanna LM, Scherer PW. A Theoretical Model of Localized Heat and Water Vapor Transport in the Human Respiratory Tract. J Biomech Eng. American Society of Mechanical Engineers; 1986;108: 19 3959548 10.1115/1.3138574

[5] Naftali S, Rosenfeld M, Wolf M, Elad D. The Air-Conditioning Capacity of the Human Nose. Ann Biomed Eng. 2005;33: 545–553. 15909660 10.1007/s10439-005-2513-4

[6] Randell SH, Boucher RC. Effective mucus clearance is essential for respiratory health. Am J Respir Cell Mol Biol. 2006;35: 20–8. 10.1165/rcmb.2006-0082SF 16528010 PMC2658694

[7] Churchill SE, Shackelford LL, Georgi JN, Black MT. Morphological variation and airflow dynamics in the human nose. Am J Hum Biol. 2004;16: 625–638. 10.1002/ajhb.20074 15495233

[8] Weiner JS. Nose Shape and Climate. Am J Phys Anthropol. 1954;12: 615–618. 14350081 10.1002/ajpa.1330120412

[9] Roseman CC, Weaver TD. Multivariate apportionment of global human craniometric diversity. Am J Phys Anthropol. 2004;125: 257–263. 10.1002/ajpa.10424 15386236

[10] Spitze K. Population structure in Daphnia obtusa: quantitative genetic and allozymic variation. Genetics. 1993;135: 367–374. Available: http://www.genetics.org/content/135/2/367 8244001 10.1093/genetics/135.2.367 PMC1205642

[11] Whitlock MC, Guillaume F. Testing for spatially divergent selection: comparing QST to FST. Genetics. 2009;183: 1055–63. 10.1534/genetics.108.099812 19687138 PMC2778959

[12] Roseman CC. Detecting interregionally diversifying natural selection on modern human cranial form by using matched molecular and morphometric data. Proc Natl Acad Sci U S A. 2004;101: 12824–9. 10.1073/pnas.0402637101 15326305 PMC516480

[13] Hubbe M, Hanihara T, Harvati K. Climate signatures in the morphological differentiation of worldwide modern human populations. Anat Rec. 2009;292: 1720–1733. 10.1002/ar.20976 19718714

[14] Guo J, Tan J, Yang Y, Zhou H, Hu S, Hashan A, et al Variation and signatures of selection on the human face. J Hum Evol. Elsevier Ltd; 2014;75: 143–152. 10.1016/j.jhevol.2014.08.001 25186351

[15] Shriver MD, Parra EJ, Dios S, Bonilla C, Norton H, Jovel C, et al Skin pigmentation, biogeographical ancestry and admixture mapping. Hum Genet. 2003;112: 387–99. 10.1007/s00439-002-0896-y 12579416

[16] Parra EJ, Kittles R a, Shriver MD. Implications of correlations between skin color and genetic ancestry for biomedical research. Nat Genet. 2004;36: S54–S60. 10.1038/ng1440 15508005

[17] Beleza S, Johnson NA, Candille SI, Absher DM, Coram MA, Lopes J, et al Genetic architecture of skin and eye color in an African-European admixed population. Spritz RA, editor. PLoS Genet. Public Library of Science; 2013;9: e1003372 10.1371/journal.pgen.1003372 23555287 PMC3605137

[18] Yang J, Benyamin B, McEvoy BP, Gordon S, Henders AK, Nyholt DR, et al Common SNPs explain a large proportion of the heritability for human height. Nat Genet. Nature Publishing Group, a division of Macmillan Publishers Limited. All Rights Reserved.; 2010;42: 565–9. 10.1038/ng.608 20562875 PMC3232052

[19] Turchin MC, Chiang CWK, Palmer CD, Sankararaman S, Reich D, Hirschhorn JN. Evidence of widespread selection on standing variation in Europe at height-associated SNPs. Nat Genet. Nature Publishing Group, a division of Macmillan Publishers Limited. All Rights Reserved.; 2012;44: 1015–9. 10.1038/ng.2368 22902787 PMC3480734

[20] Jablonski NG. the Evolution of Human Skin and Skin Color. Annu Rev Anthropol. 2004;33: 585–623.

[21] Lamason RL, Mohideen M-APK, Mest JR, Wong AC, Norton HL, Aros MC, et al SLC24A5, a putative cation exchanger, affects pigmentation in zebrafish and humans. Science. 2005;310: 1782–6. 10.1126/science.1116238 16357253

[22] Norton HL, Kittles RA, Parra E, McKeigue P, Mao X, Cheng K, et al Genetic evidence for the convergent evolution of light skin in Europeans and East Asians. Mol Biol Evol. 2007;24: 710–22. 10.1093/molbev/msl203 17182896

[23] Jablonski NG, Chaplin G. The evolution of human skin coloration. J Hum Evol. 2000;39: 57–106. 10.1006/jhev.2000.0403 10896812

[24] Gower JC. Generalized procrustes analysis. Psychometrika. 1975;40: 33–51.

[25] Claes P, Liberton DK, Daniels K, Rosana KM, Quillen EE, Pearson LN, et al Modeling 3D Facial Shape from DNA. Luquetti D, editor. PLoS Genet. Public Library of Science; 2014;10: e1004224 10.1371/journal.pgen.1004224 24651127 PMC3961191

[26] Ferrario VF, Mian F, Peretta R, Rosati R, Sforza C. Three-dimensional computerized anthropometry of the nose: Landmark representation compared to surface analysis. Cleft Palate-Craniofacial J. 2007;44: 278–285. 10.1597/06-021 17477754

[27] Nucara A, Pietrafesa M, Rizzo G, Scaccianoce G. Handbook of Anthropometry. Handb Anthr. 2012; 91–114.

[28] Leinonen T, McCairns RJS, O’Hara RB, Merilä J. Q(ST)-F(ST) comparisons: evolutionary and ecological insights from genomic heterogeneity. Nat Rev Genet. Nature Publishing Group, a division of Macmillan Publishers Limited. All Rights Reserved.; 2013;14: 179–90. 10.1038/nrg3395 23381120

[29] Wright S. The Genetical Structure Of Populations. Ann Eugen. 1949;15: 323–354. 10.1111/j.1469-1809.1949.tb02451.x 24540312

[30] Lande R. Neutral Theory of Quantitative Genetic Variance in an Island Model with Local Extinction and Colonization. Evolution (N Y). 1992;46: 381–389. 10.1111/j.1558-5646.1992.tb02046.x 28564025

[31] Whitlock MC. Evolutionary inference from QST. Molecular Ecology. 2008 pp. 1885–1896. 10.1111/j.1365-294X.2008.03712.x 18363667

[32] Brommer JE. Whither Pst? The approximation of Qst by Pst in evolutionary and conservation biology. J Evol Biol. 2011;24: 1160–8. 10.1111/j.1420-9101.2011.02268.x 21457173

[33] Leinonen T, Cano JM, Mäkinen H, Merilä J. Contrasting patterns of body shape and neutral genetic divergence in marine and lake populations of threespine sticklebacks. J Evol Biol. 2006;19: 1803–12. 10.1111/j.1420-9101.2006.01182.x 17040377

[34] Perry GH, Foll M, Grenier J-C, Patin E, Nédélec Y, Pacis A, et al Adaptive, convergent origins of the pygmy phenotype in African rainforest hunter-gatherers. Proc Natl Acad Sci U S A. 2014;111: E3596–603. 10.1073/pnas.1402875111 25136101 PMC4156716

[35] Relethford JH. Craniometric variation among modern human populations. Am J Phys Anthropol. 1994;95: 53–62. 10.1002/ajpa.1330950105 7527996

[36] Lynch M, Walsh B. Genetics and analysis of quantitative traits Sunderland, MA: Sinauer Associates, Inc; 1998.

[37] Yang J, Lee SH, Goddard ME, Visscher PM. GCTA: a tool for genome-wide complex trait analysis. Am J Hum Genet. 2011;88: 76–82. 10.1016/j.ajhg.2010.11.011 21167468 PMC3014363

[38] Sabeti PC, Varilly P, Fry B, Lohmueller J, Hostetter E, Cotsapas C, et al Genome-wide detection and characterization of positive selection in human populations. Nature. 2007;449: 913–918. 10.1038/nature06250 17943131 PMC2687721

[39] Darvasi A, Shifman S. The beauty of admixture. Nat Genet. 2005;37: 118–119. 10.1038/ng0205-118 15678141

[40] Zaitlen N, Pasaniuc B, Sankararaman S, Bhatia G, Zhang J, Gusev A, et al Leveraging population admixture to characterize the heritability of complex traits. Nat Genet. Nature Publishing Group, a division of Macmillan Publishers Limited. All Rights Reserved.; 2014;46: 1356–1362. 10.1038/ng.3139 25383972 PMC4244251

[41] Yokley TR. Ecogeographic variation in human nasal passages. Am J Phys Anthropol. 2009;138: 11–22. 10.1002/ajpa.20893 18623075

[42] Noback ML, Harvati K, Spoor F. Climate-related variation of the human nasal cavity. Am J Phys Anthropol. 2011;145: 599–614. 10.1002/ajpa.21523 21660932

[43] Maddux SD, Yokley TR, Svoma BM, Franciscus RG. Absolute humidity and the human nose: A reanalysis of climate zones and their influence on nasal form and function. Am J Phys Anthropol. 2016;161: 309–320. 10.1002/ajpa.23032 27374937

[44] Keck T, Lindemann J. Numerical simulation and nasal air-conditioning. GMS Curr Top Otorhinolaryngol Head Neck Surg. 2010;9: Doc08 10.3205/cto000072 22073112 PMC3199825

[45] Inthavong K, Tian ZF, Tu JY. CFD Simulations on the Heating Capability in a Human Nasal Cavity. Rhinology. 2007; 842–847.

[46] Irmak MK, Korkmaz A, Erogul O. Selective brain cooling seems to be a mechanism leading to human craniofacial diversity observed in different geographical regions. Med Hypotheses. 2004;63: 974–979. 10.1016/j.mehy.2004.05.003 15504564

[47] Maloney SK, Mitchell D, Blache D. The contribution of carotid rete variability to brain temperature variability in sheep in a thermoneutral environment. Am J Physiol Regul Integr Comp Physiol. 2007;292: R1298–305. 10.1152/ajpregu.00275.2006 17082355

[48] Mitchell D, Maloney SK, Jessen C, Laburn HP, Kamerman PR, Mitchell G, et al Adaptive heterothermy and selective brain cooling in arid-zone mammals. Comp Biochem Physiol Part B Biochem Mol Biol. 2002;131: 571–585. 10.1016/s1096-4959(02)00012-x 11923074

[49] Hetem RS, Strauss WM, Fick LG, Maloney SK, Meyer LCR, Fuller A, et al Selective brain cooling in Arabian oryx (Oryx leucoryx): a physiological mechanism for coping with aridity? J Exp Biol. 2012;215: 3917–24. 10.1242/jeb.074666 22899527

[50] Blix AS, Walløe L, Folkow LP. Regulation of brain temperature in winter-acclimatized reindeer under heat stress. J Exp Biol. 2011;214: 3850–6. 10.1242/jeb.057455 22031750

[51] Crandall CG, Brothers RM, Zhang R, Brengelmann GL, Covaciu L, Jay O, et al Comments on point:counterpoint: humans do/do not demonstrate selective brain cooling during hyperthermia. J Appl Physiol. American Physiological Society; 2011;110: 575–80. 10.1152/japplphysiol.01375.2010 21304015

[52] Cabanac M. Selective brain cooling in humans: “fancy” or fact? FASEB J. 1993;7: 1143-1146-1147. 8375612 10.1096/fasebj.7.12.8375612

[53] Puts DA. Beauty and the beast: mechanisms of sexual selection in humans. Evol Hum Behav. Elsevier; 2010;31: 157–175.

[54] van Doorn GS, Edelaar P, Weissing FJ. On the origin of species by natural and sexual selection. Science. 2009;326: 1704–1707. 10.1126/science.1181661 19965377

[55] Bigham AW, Mao X, Mei R, Brutsaert T, Wilson MJ, Julian CG, et al Identifying positive selection candidate loci for high-altitude adaptation in Andean populations. Hum Genomics. 2009;4: 79–90. 10.1186/1479-7364-4-2-79 20038496 PMC2857381

[56] Wu T, Li S, Ward MP. Tibetans at extreme altitude. Wilderness Environ Med. 2005;16: 47–54. 15813148 10.1580/pr04-04.1

[57] Paternoster L, Zhurov AI, Toma AM, Kemp JP, St Pourcain B, Timpson NJ, et al Genome-wide association study of three-dimensional facial morphology identifies a variant in PAX3 associated with nasion position. Am J Hum Genet. Elsevier; 2012;90: 478–85. 10.1016/j.ajhg.2011.12.021 22341974 PMC3309180

[58] Liu F, van der Lijn F, Schurmann C, Zhu G, Chakravarty MM, Hysi PG, et al A genome-wide association study identifies five loci influencing facial morphology in Europeans. Gibson G, editor. PLoS Genet. Public Library of Science; 2012;8: e1002932 10.1371/journal.pgen.1002932 23028347 PMC3441666

[59] Shaffer JR, Orlova E, Lee MK, Leslie EJ, Raffensperger ZD, Heike CL, et al Genome-Wide Association Study Reveals Multiple Loci Influencing Normal Human Facial Morphology. Barsh GS, editor. PLOS Genet. Public Library of Science; 2016;12: e1006149 10.1371/journal.pgen.1006149 27560520 PMC4999139

[60] Adhikari K, Fuentes-Guajardo M, Quinto-Sánchez M, Mendoza-Revilla J, Camilo Chacón-Duque J, Acuña-Alonzo V, et al A genome-wide association scan implicates DCHS2, RUNX2, GLI3, PAX1 and EDAR in human facial variation. Nat Commun. Nature Research; 2016;7: 11616. 10.1038/ncomms11616 PMC4874031 27193062

[61] Beleza S, Campos J, Lopes J, Araújo II, Hoppfer Almada A, Correia e Silva A, et al The Admixture Structure and Genetic Variation of the Archipelago of Cape Verde and Its Implications for Admixture Mapping Studies. PLoS One. 2012;7. 10.1371/journal.pone.0051103 PMC3511383 23226471

[62] Illumina Inc. “TOP/BOT” strand and “A/B” allele—A guide to Illumina’s method for determining Strand and Allele for the GoldenGate and Infinium Assays. (Technical Note) [Internet]. 2006. Available: http://www.illumina.com/documents/products/technotes/technote_topbot.pdf

[63] Purcell S, Neale B, Todd-Brown K, Thomas L, Ferreira MAR, Bender D, et al PLINK: a tool set for whole-genome association and population-based linkage analyses. Am J Hum Genet. 2007;81: 559–75. 10.1086/519795 17701901 PMC1950838

[64] Chang CC, Chow CC, Tellier LC, Vattikuti S, Purcell SM, Lee JJ. Second-generation PLINK: rising to the challenge of larger and richer datasets. Gigascience. BioMed Central Ltd; 2015;4: 7 10.1186/s13742-015-0047-8 25722852 PMC4342193

[65] Candille SI, Absher DM, Beleza S, Bauchet M, McEvoy B, Garrison NA, et al Genome-wide association studies of quantitatively measured skin, hair, and eye pigmentation in four European populations. PLoS One. Public Library of Science; 2012;7: e48294 10.1371/journal.pone.0048294 23118974 PMC3485197

[66] Storz JF. Contrasting patterns of divergence in quantitative traits and neutral DNA markers: analysis of clinal variation. Mol Ecol. 2002;11: 2537–2551. 12453238 10.1046/j.1365-294x.2002.01636.x

[67] He Y, Li R, Wang J, Blanchet S, Lek S. Morphological Variation Among Wild Populations of Chinese Rare Minnow (Gobiocypris rarus): Deciphering the Role of Evolutionary Processes. Zoolog Sci. 2013;30: 475–83. 10.2108/zsj.30.475 23721472

[68] Weir BS, Cockerham CC. Estimating F-statistics for the analysis of population structure. Evolution (N Y). 1984;38: 1358–1370. 10.1111/j.1558-5646.1984.tb05657.x 28563791

[69] Browning SR, Browning BL. Population structure can inflate SNP-based heritability estimates. Am J Hum Genet. 2011;89: 191–193. 10.1016/j.ajhg.2011.05.025 21763486 PMC3135810

[70] Goddard ME, Lee SH, Yang J, Wray NR, Visscher PM. Response to browning and browning. Am J Hum Genet. 2011;89: 193–195.

[71] Baran Y, Pasaniuc B, Sankararaman S, Torgerson DG, Gignoux C, Eng C, et al Fast and accurate inference of local ancestry in Latino populations. Bioinformatics. 2012;28: 1359–1367. 10.1093/bioinformatics/bts144 22495753 PMC3348558

[72] Halder I, Shriver MD. Measuring and using admixture to study the genetics of complex diseases. Hum Genomics. BioMed Central Ltd; 2003;1: 52 10.1186/1479-7364-1-1-52 15601533 PMC3525000

[73] Kahle D, Wickham H. ggmap: Spatial Visualization with. R J. 2013;5: 144–161. Available: http://journal.r-project.org/archive/2013-1/kahle-wickham.pdf

[74] R Core Team. R: A language and environment for statistical computing R Foundation for Statistical Computing, Vienna, Austria 2013.

[75] Harris I, Jones PD, Osborn TJ, Lister DH. Updated high-resolution grids of monthly climatic observations—the CRU TS3.10 Dataset. Int J Climatol. John Wiley & Sons, Ltd; 2014;34: 623–642.

[76] TOMS Science Team (1996). TOMS Earth Probe UV Reflectivity Monthly L3 Global 1 deg x 1.25 deg Lat/Lon Grid V008, version 008, Greenbelt, MD, Goddard Earth Sciences Data and Information Services Center (GES DISC) [Internet]. Available: http://disc.sci.gsfc.nasa.gov/datacollection/TOMSEPL3mref_008.html

[77] Michna P, Woods M. RNetCDF—A Package for Reading and Writing NetCDF Datasets. R J. 2013;5: 29–35.

[78] Hijmans RJ, Etten J Van. raster: Geographic analysis and modeling with raster data. R package version 2.0–12. [Internet]. 2012. Available: http://cran.r-project.org/package=raster

[79] Rousset F, Ferdy J-B. Testing environmental and genetic effects in the presence of spatial autocorrelation. Ecography (Cop). 2014;37: 781–790.

[80] Claes P, Reijniers J, Shriver MD, Snyders J, Suetens P, Nielandt J, et al An investigation of matching symmetry in the human pinnae with possible implications for 3D ear recognition and sound localization. J Anat. 2015;226: 60–72. 10.1111/joa.12252 25382291 PMC4313899

[81] Pinheiro J, Bates D, DebRoy S, Sarkar D, Team TRC. nlme: Linear and Nonlinear Mixed Effects Models. October. 2013 pp. 1–3.

