# Correction: Investigating the case of human nose shape and climate adaptation

**DOI:** 10.1371/journal.pgen.1007207

**Published:** 2018-01-31

**Authors:** Arslan A. Zaidi, Brooke C. Mattern, Peter Claes, Brian McEvoy, Cris Hughes, Mark D. Shriver

The fourth author's name is spelled incorrectly. The correct name is: Brian McEvoy. The correct citation is: Zaidi AA, Mattern BC, Claes P, McEvoy B, Hughes C, Shriver MD (2017) Investigating the case of human nose shape and climate adaptation. PLoS Genet 13(3): e1006616. doi.org/10.1371/journal.pgen.1006616
