# Supplementary figures and images for: Investigating the case of human nose shape and climate adaptation
Source: PLoS Genet. 2017 Mar 16;13(3):e1006616. doi: 10.1371/journal.pgen.1006616 (PMC5354252; doi:10.1371/journal.pgen.1006616)

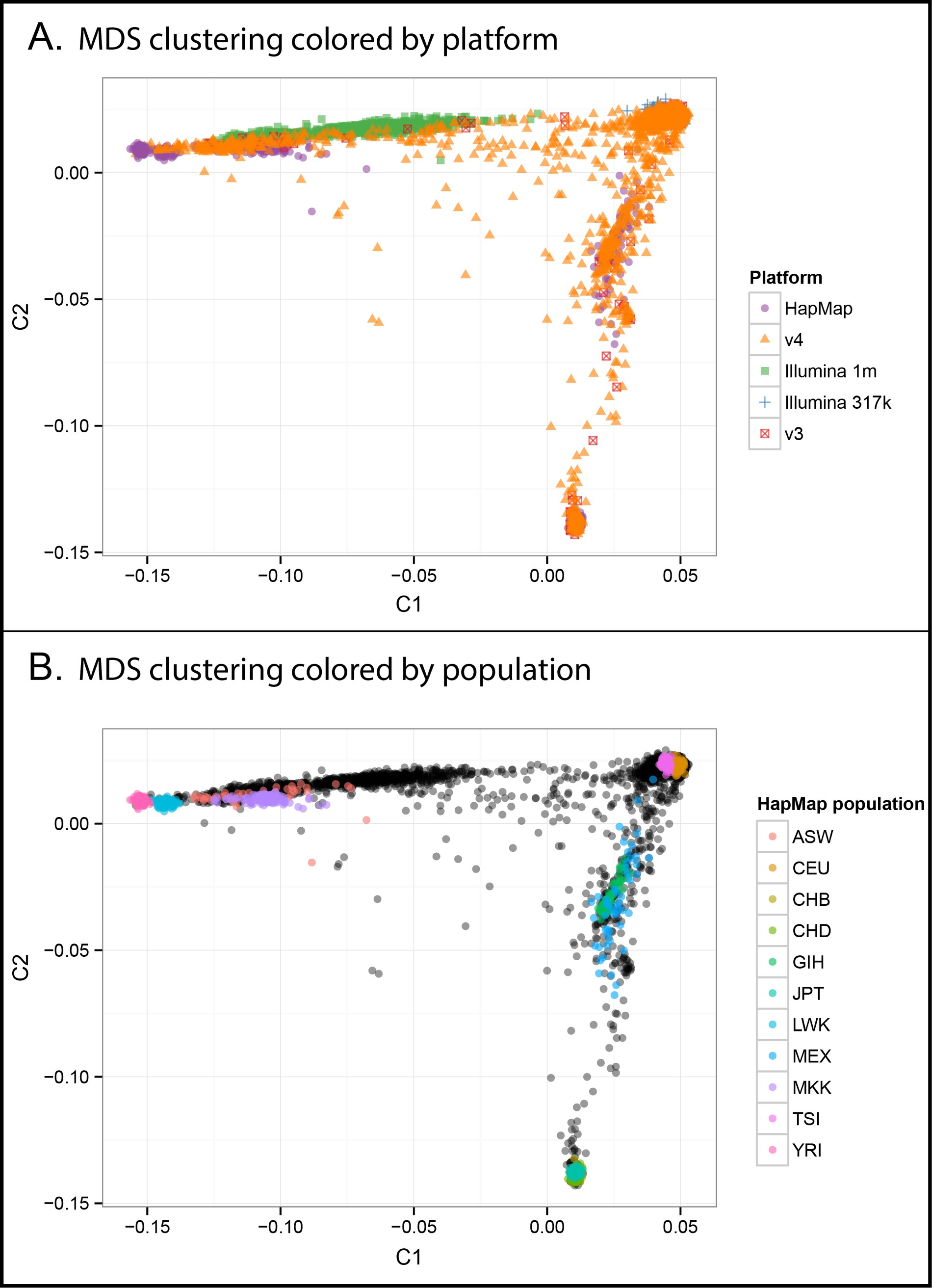

Supplement: S1 Fig — MDS plots (k = 2) showing clustering of genotyped individuals by A) genotyping platform and B) by ancestry. In B) the genotyped individuals from the study set are shown in black, while the HapMap samples are shown in color for reference. The plot shows that individuals cluster by ancestry and not by genotyping platform. Thus, batch effects due to genotyping platform are unlikely to lead to artifacts in ancestry inference. (TIF) [file pgen.1006616.s001.tif]

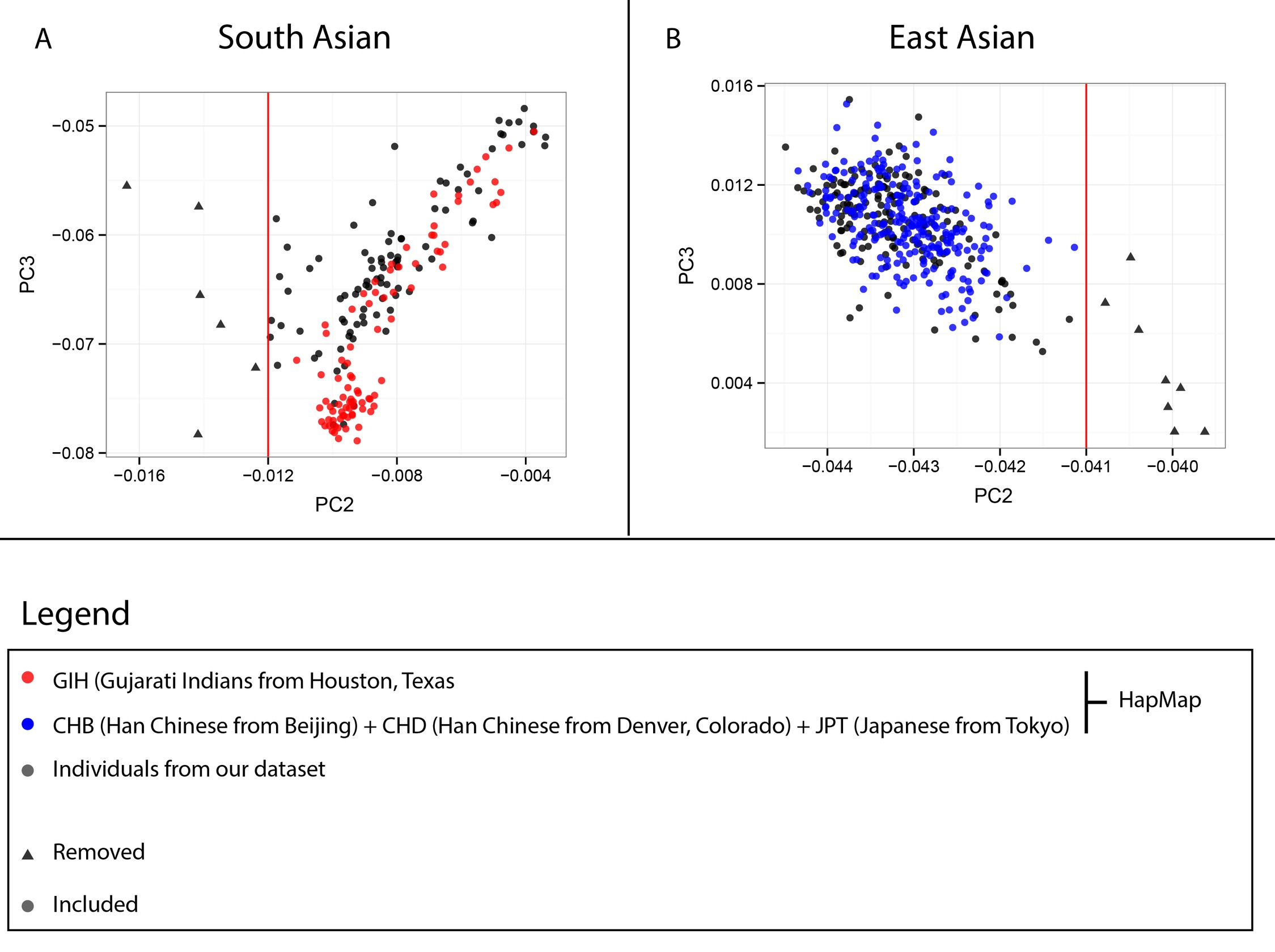

Supplement: S2 Fig — PCA plots showing clustering of genotyped individuals by A) South Asian and B) East Asian ancestry around HapMap reference samples. The colored points are reference samples from the HapMap dataset and the grey points are individuals from our dataset. A) South Asian individuals are shown with the HapMap GIH samples and B) East Asian individuals are shown with the combined East Asian sample (CHB+JPT+CHD) from the HapMap dataset. The red lines indicate the cutoff for removing individuals who appear to cluster far away from the main cluster. The individuals who were removed, based on clustering, are shown as triangles, whereas all other individuals are shown as circles. (TIF) [file pgen.1006616.s002.tif]

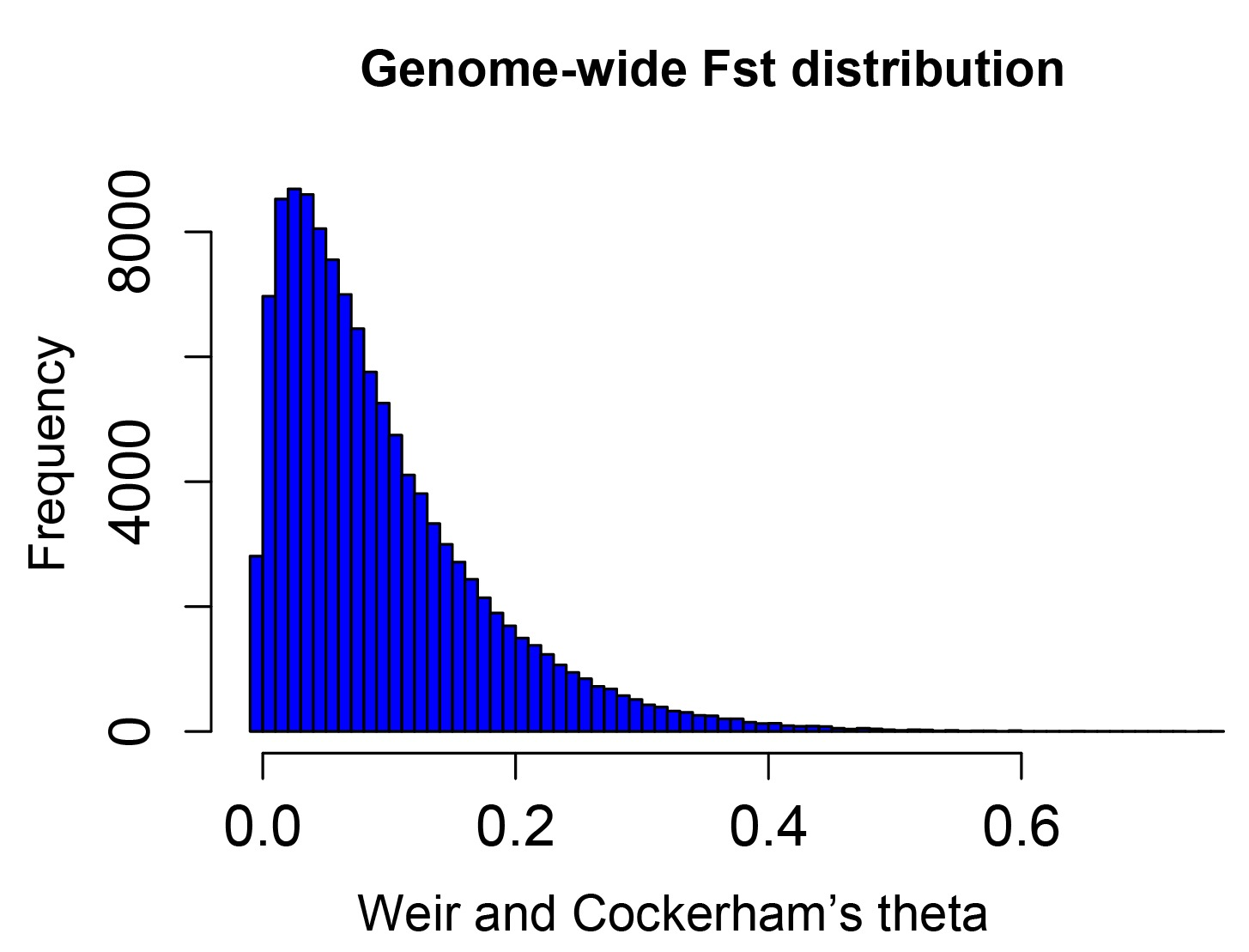

Supplement: S3 Fig — The distribution was generated from 118,420 autosomal SNPs using Weir and Cockerham’s θ. (TIF) [file pgen.1006616.s003.tif]

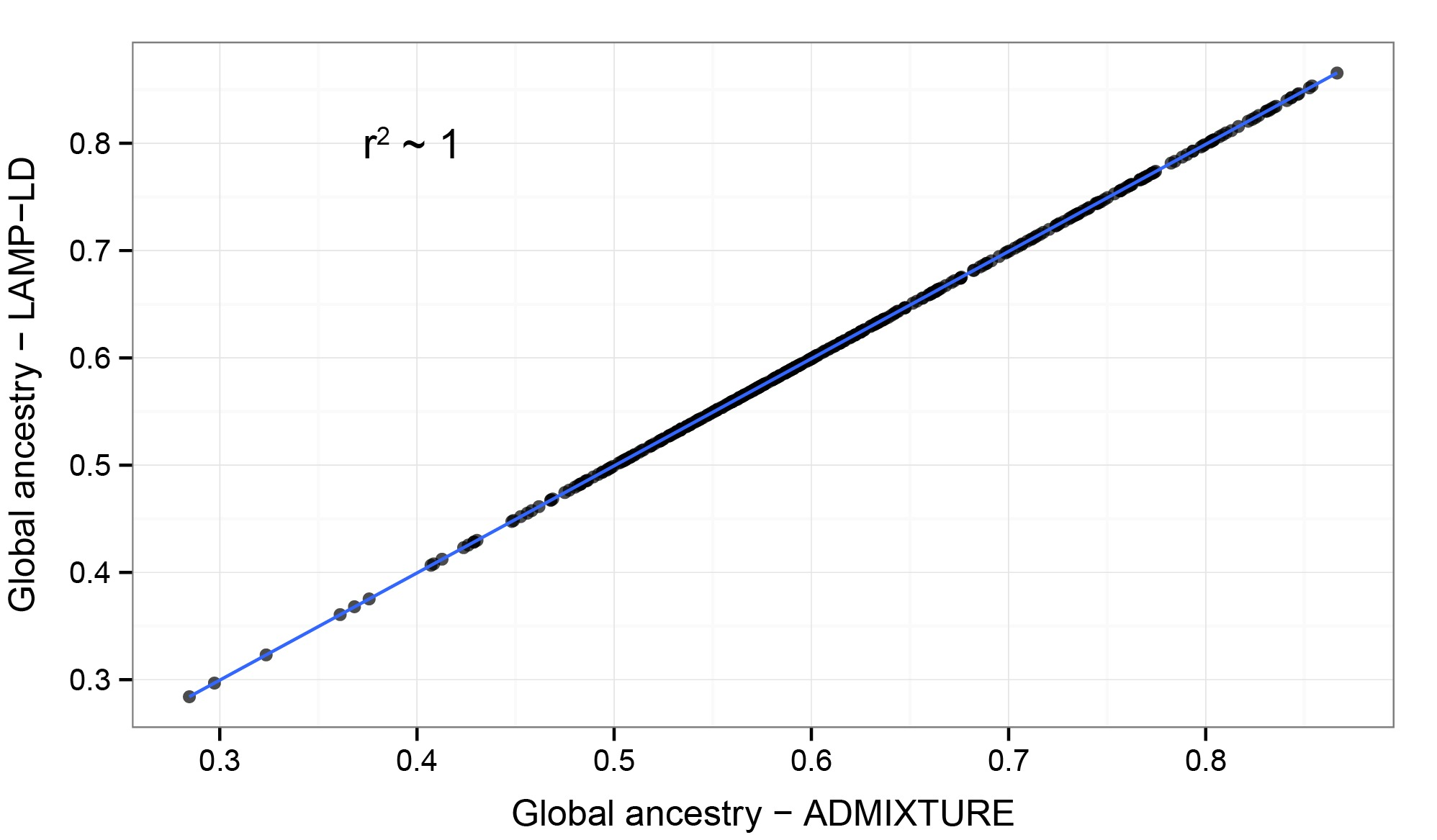

Supplement: S4 Fig — ADMIXTURE-based ancestry estimates were calculated using a k = 2 assumption. LAMP-LD-based ancestry estimates were calculating the mean local ancestry across 623,625 autosomal SNPs. (TIF) [file pgen.1006616.s004.tif]

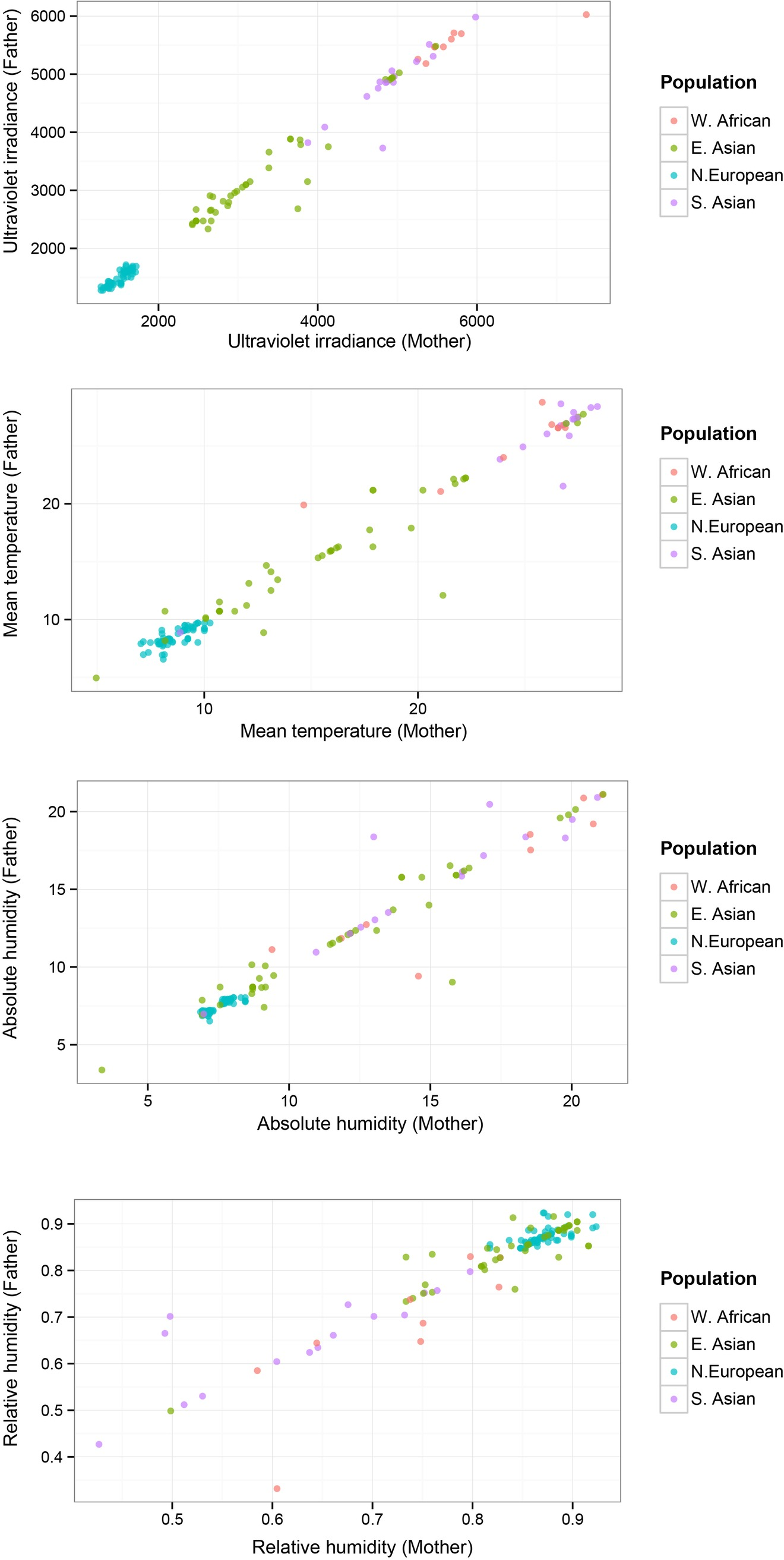

Supplement: S5 Fig — A) Mean annual temperature is measured in degrees Celsius. B) Annual aridity index is a ratio. C) UVB irradiance is measured in J/m2. (TIF) [file pgen.1006616.s005.tif]

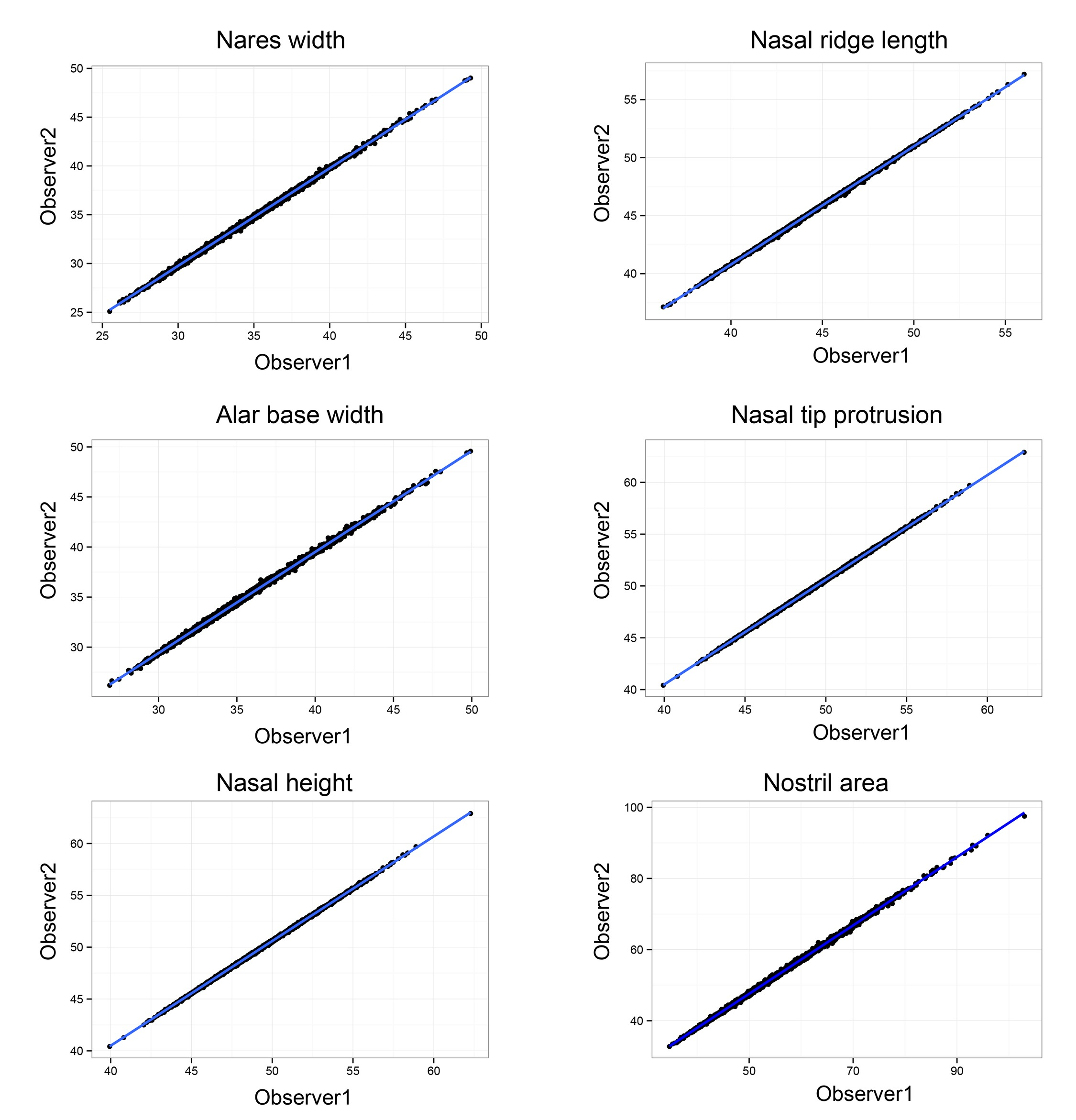

Supplement: S6 Fig — (TIF) [file pgen.1006616.s006.tif]

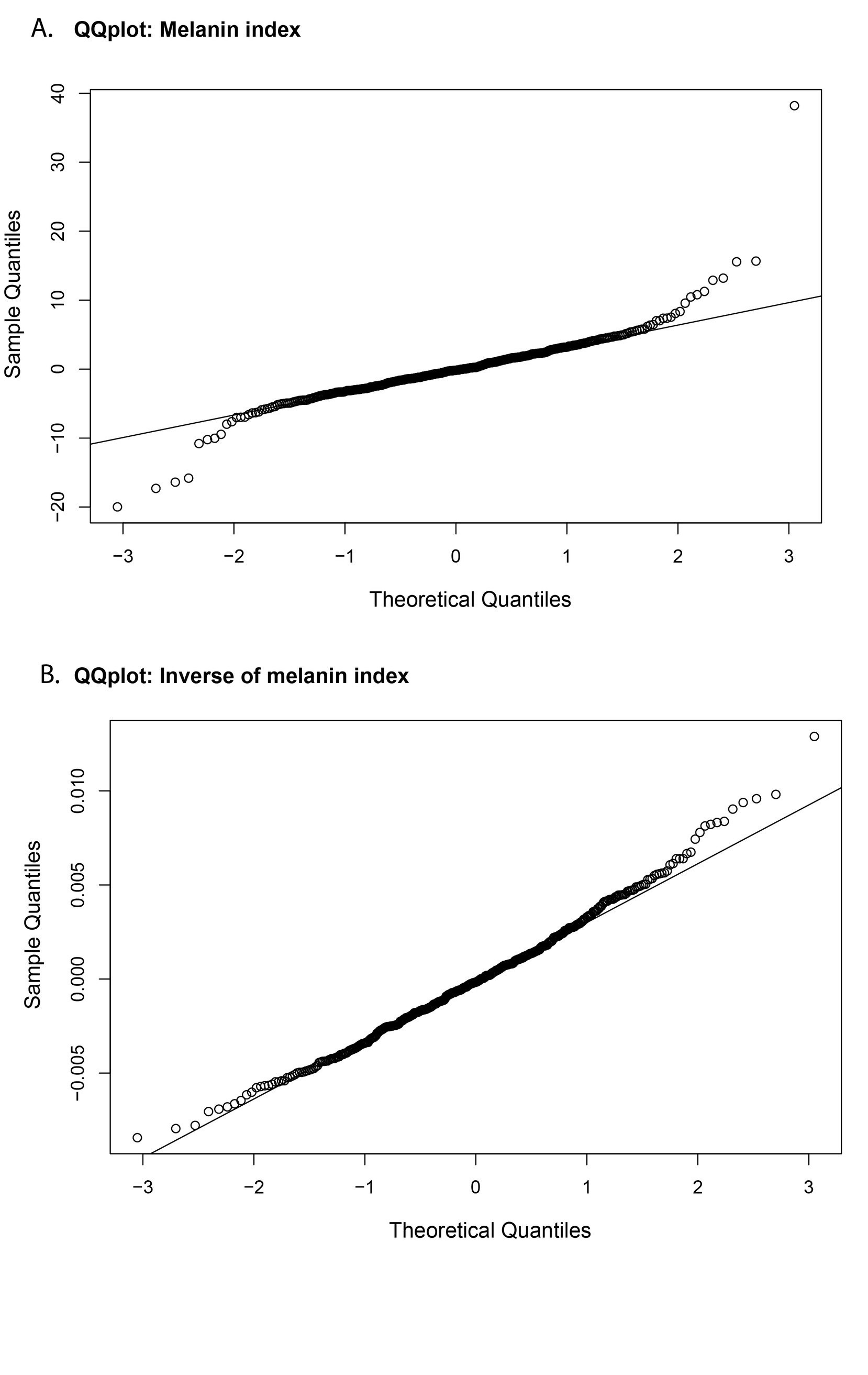

Supplement: S7 Fig — QQplots showing that the residuals for A) the inverse of melanin index are more normally distributed than B) untransformed melanin index. The points are residuals from linear models where population, sex, age, and BMI were used as predictors. (TIF) [file pgen.1006616.s007.tif]
