# Supplementary material for: Investigating the case of human nose shape and climate adaptation
Source: PLoS Genet. 2017 Mar 16;13(3):e1006616. doi: 10.1371/journal.pgen.1006616 (PMC5354252; doi:10.1371/journal.pgen.1006616)
Supplement: S1 Table — (DOCX) [file pgen.1006616.s008.docx]

Table S1: ANOVA table for each phenotype

|  | Height | | | |
| --- | --- | --- | --- | --- |
| Sum | SS | Df | F | P |
| Population | 5877.0 | 3 | 40.89 | <2e-16 |
| Sex | 21872.8 | 1 | 456.51 | <2e-16 |
| Age | 1.8 | 1 | 0.04 | 0.847 |
| BMI | 58.7 | 1 | 1.22 | 0.269 |
| Residuals | 22471.3 | 469 |  |  |
|  | | | | |
|  | Melanin Index | | | |
|  | SS | Df | F | P |
| Population | 34062 | 3 | 597.50 | <2e-16 |
| Sex | 187 | 1 | 9.83 | 0.002 |
| Age | 3 | 1 | 0.16 | 0.691 |
| BMI | 1 | 1 | 0.06 | 0.803 |
| Residuals | 8171 | 430 |  |  |
|  | | | | |
|  | Nares Width | | | |
|  | SS | Df | F | P |
| Population | 3207.0 | 3 | 216.11 | <2e-16 |
| Sex | 1678.6 | 1 | 339.35 | <2e-16 |
| Age | 72.7 | 1 | 14.70 | 1.43e-04 |
| BMI | 59.1 | 1 | 11.95 | 5.97e-04 |
| Residuals | 2319.9 | 469 |  |  |
|  | | | | |
|  | Alar Base Width | | | |
|  | SS | Df | F | P |
| Population | 2643.9 | 3 | 195.14 | <2e-16 |
| Sex | 1065.7 | 1 | 235.97 | <2e-16 |
| Age | 139.5 | 1 | 30.88 | 4.61e-08 |
| BMI | 83.8 | 1 | 18.55 | 2.02e-05 |
| Residuals | 2118.2 | 469 |  |  |
|  | | | | |
|  | Nasal Height | | | |
|  | SS | Df | F | P |
| Population | 19.84 | 3 | 6.61 | 0.301 |
| Sex | 1096.2 | 1 | 202.37 | <2e-16 |
| Age | 25.7 | 1 | 4.75 | 0.030 |
| BMI | 6.6 | 1 | 1.22 | 0.270 |
| Residuals | 2540.5 | 469 |  |  |
|  | | | | |
|  | Nasal Ridge Length | | | |
|  | SS | Df | F | P |
| Population | 174.8 | 3 | 9.80 | 2.79e-06 |
| Sex | 1112.7 | 1 | 187.10 | <2e-16 |
| Age | 115.7 | 1 | 19.45 | 1.28e-05 |
| BMI | 0.2 | 1 | 0.03 | 0.861 |
| Residuals | 2789.2 | 469 |  |  |
|  | | | | |
|  | Nasal Tip Protrusion | | | |
|  | SS | Df | F | P |
| Population | 391.9 | 3 | 101.63 | <2e-16 |
| Sex | 158.5 | 1 | 123.29 | <2e-16 |
| Age | 21.5 | 1 | 16.70 | 5.26E-05 |
| BMI | 5.8 | 1 | 4.48 | 0.035 |
| Residuals | 602.9 | 469 |  |  |
|  | | | | |
|  | External Area | | | |
|  | SS | Df | F | P |
| Population | 2655003 | 3 | 54.11 | <2e-16 |
| Sex | 9286528 | 1 | 567.75 | <2e-16 |
| Age | 406030 | 1 | 24.82 | 8.85e-07 |
| BMI | 55136 | 1 | 3.37 | 0.067 |
| Residuals | 7671297 | 469 |  |  |
|  | | | | |
|  | Nostril Area | | | |
|  | SS | Df | F | P |
| Population | 6169.6 | 3 | 55.00 | <2e-16 |
| Sex | 12014.9 | 1 | 321.33 | <2e-16 |
| Age | 2103.7 | 1 | 56.26 | 3.20E-13 |
| BMI | 280.6 | 1 | 7.50 | 0.006 |
| Residuals | 17536.2 | 469 |  |  |
|  | | | | |
